# Leaf tissue metabolomics fingerprinting of *Citronella gongonha* Mart. by ^1^H HR-MAS NMR

**DOI:** 10.1038/s41598-022-22708-w

**Published:** 2022-10-21

**Authors:** Sher Ali, Gul Badshah, Umar Ali, Muhammad Siddique Afridi, Anwar Shamim, Ajmir Khan, Frederico Luiz Felipe Soares, Leociley Rocha Alencar Menezes, Vanessa Theodoro Rezende, Andersson Barison, Carlos Augusto Fernandes de Oliveira, Fernando Gustavo Tonin

**Affiliations:** 1 Department of Chemistry, NMR Center, Federal University of Paraná (UFPR), Curitiba, PR 81530-000 Brazil; 2 Faculty of Animal Science and Food Engineering (FZEA), Department of Food Engineering, University of São Paulo (USP), Pirassununga, SP 13635-900 Brazil; 3 Laboratory of Polymers and Catalysis (LaPoCa), Department of Chemistry, Federal University of Paraná (UFPR), Curitiba, PR 81530-000 Brazil; 4 Department of Physics, University of Malakand (UoM), Dir (L), 18800 KPK Pakistan; 5 Department of Plant Pathology, Federal University of Lavras (UFLA), 3037, Lavras, 37200-900 Brazil; 6 Group of Medicinal and Biological Chemistry, University of São Paulo-São Carlos Institute of Chemistry (IQSC-USP), São Carlos, SP 13566-590 Brazil; 7 School of Packaging, Michigan State University (MSU), 448 Wilson Road, East Lansing, MI 48824-1223 USA; 8 Data Science in Chemistry Laboratory, Department of Chemistry, Federal University of Paraná (UFPR), Curitiba, PR 81530-000 Brazil; 9 Faculty of Veterinary and Animal Science (FMVZ), Department of Animal Science, University of São Paulo (USP), Pirassununga, SP 13635-900 Brazil; 10 Faculty of Animal Science and Food Engineering (FZEA), Department of Biosystems Engineering, University of São Paulo (USP), Pirassununga, SP 13635-900 Brazil

**Keywords:** NMR spectroscopy, Metabolomics

## Abstract

This research characterizes key metabolites in the leaf from *Citronella gongonha* Martius (Mart.) Howard (Cardiopteridaceae). All metabolites were assessed in intact leaf tissue by proton (^1^H) high-resolution magic angle spinning (HR-MAS) nuclear magnetic resonance (NMR) spectroscopy integrated with the principal component analysis (PCA) to depict molecular association with the seasonal change. The major ‘known unknown’ metabolites detected in ^1^H HR-MAS NMR were derivatives of flavonoid, polyphenolic and monoterpenoid compounds such as kaempferol-3-*O*-dihexoside, caffeoyl glucoside (**2**), 3-*O*-caffeoylquinic acid (**3**), 5-*O*-caffeoylquinic acid (**4**), kingiside (**5**), 8-epi-kingisidic acid (**6**), (7*α*)-7-*O*-methylmorroniside (**7**), (7*β*)-7-*O*-methylmorroniside (**8**) and alpigenoside (**9**) together with the universally occurring sucrose (**10**), *α*-glucoses (**11, 12**), alanine (**13**), and fatty (linolenic) acid (**14**). Several of the major metabolites (1, 2–9) were additionally confirmed by liquid chromatography tandem mass spectrometry (LC–MS/MS). In regard with the PCA results, metabolites 1, 2–9 and 14 were influenced by seasonal variation and/or from further (a) biotic environmental conditions. The findings in this work indicate that *C. gongonha* Mart. is an effective medicinal plant by preserving particularly compounds 2, 3–9 in abundant amounts. Because of close susceptibility with seasonal shift and ecological trends, further longitudinal studies are needed to realize the physiology and mechanism involved in the production of these and new metabolites in this plant under controlled conditions. Also, future studies are recommended to classify different epimers, especially of the phenolics and monoterpenoids in the given plant.

## Introduction

*Citronella gongonha* Mart. Howard from Cardiopteridaceae family is a classical and taxonomically known Brazilian plant. This family represents total of 43 species, and six genera, of which one of the largest is *Citronella* that contains *C. gongonha* Mart. in amongst 21 species. As broadly distributed, this species can be found in several Brazilian regions; Curitiba-Paraná, Irati and Alto Rio Grande-Minas Gerais^1,2^. Some alternative names for this species are; *Congonha*, *Orange-plum*, *Villaresia cuspidata* Miers., *Cassine gongonha* Mart., *Laranjeira-do-banhado*, *Ilex gongonha* (Mart.) D. Don, *Myginda gongonha* (Mart.) DC., *Villaresia congonha* Miers, and *Villaresia gongonha* (Mart.) Miers (“http://floradigital.ufsc.br” and “Useful Tropical Plants”). With a deciduous and non-flexible wood or shrubby nature, such plant reaches almost eight meters tall. Possessing smooth and marginal spiny leaves, *C. gongonha* produces scented and white-purple flowers that end up with oval-shaped brownish fruits. Due to the leaf morphology, this plant has close similarity to other phytotherapeutics that are usually consumed in Brazil and other countries^3^. In terms of chemically important natural products (NPs), *C. gongonha* Mart. is one of the species that lacks chemical notion.

NPs have been studied since antiquity, and their convincing nature in innovative medicines is inevitable. As products of the primary and secondary metabolism in plant, NPs are multiclass organic small metabolites that can benefit drug and medicinal chemistry. In addition to the competent nature in energy and environmental fitness^4^, several metabolites have proven high medicinal values^5^. Amongst a multitude of metabolites, the medicinal relevance has mostly attributed to terpenoids including iridoids and secoiridoids^6,7^, flavonoid—e.g., kaempferol^8,9^, and several derivatives of caffeic acid^10–12^. With respect to the characterization, plant metabolites have commonly been separated, purified and detected through the popular liquid chromatography (LC), spectrometric (MS) and spectroscopic (NMR) tools, yet their integrated modes are also on record^4,13–15^. As routinely used, each of these tools has (dis)advantages, pertinency, reproducibility, selectivity to sensitivity, detection range, sample type and preparation methods^15^. However, due to great benefits, state-of-the-art high-resolution NMR and MS are the primary and most commonly used tools in metabolomics investigation^14,15^. If compare to MS, NMR is more reproducible that can non-selectively transform the atomic scale data from the sample in liquid, solid and semisolid nature to a highly informative insight^15^. Metabolomics is an emerged approach that has to do with the inclusive breadth of small metabolites in a system metabolome of plant and or other origin including humans. In this respect, a number of methods have been tailored to detach, refine and make metabolites more viable for metabolomics analysis by MS, NMR, and related tools^16^. However, extraction methods cannot be easily available, and if present, they can be selective and compromised to the reliability of molecules. In line to keep molecular consistency, it is now possible to detect metabolites within an intact tissue through ^1^H HR-MAS NMR spectroscopy^3,17,18^.

HR-MAS NMR is an emerged hybrid tool designed with the liquid- and solid-state skills, has a decisive role in tracking metabolites directly in composite semisolid tissues in negligible amount that made tissue metabolomics attractive^3,17,19,20^. In recent years, ^1^H HR-MAS NMR-based tissue metabolomics has greatly assisted to tracing environmental perturbations on the plant metabolome^3,17^. It is worth noting to understand that the leaf tissue has a microscopically controlled heterogenous microenvironment with plentiful water. Whereas, due to partially compact microenvironment, molecules remain immobile and present in closed proximities with superior atomic anisotropic interactions of several thousands of hertz (Hz) in magnitude. Therefore, these factors cause uninterpretable and substandard ^1^H HR-MAS NMR signals (broadened signals)^3,21^. Subduing such unsatisfactory interactions, molecular mobility and water in the sample, can allow one to attain ^1^H HR-MAS NMR spectra of adequate resolution. Of note, ^1^H HR-MAS NMR capably spots molecules at the solvent interface via solution-state experiment, whilst the unsolicited atomic interactions are averaged by solid-state MAS technique^22^. ^1^H HR-MAS NMR-based tissue metabolomics provides enhanced advantages, involving molecular integrity, hence fingerprinting by this tool has satisfied researchers, dealing with diverse objectives^3,17,18,20,23^.

Leaf tissue metabolomics by ^1^H HR-MAS NMR combined with the PCA tool, in this work, has been implemented to cover the metabolic fingerprints and their relation with the seasonal alteration in a limitedly explored *C. gongona* Mart. High-resolution two-dimensional (2D) NMR in solution-state has also been presented, aiming at the chemical structures elucidation of the metabolites detected in ^1^H HR-MAS NMR. As a supplementary tool, the LC–MS/MS has also been used to confirm chemical structures of the major metabolites—e.g., compounds **1**, **2–9** (Fig. 2), respectively.

## Results and discussion

### Leaf tissue fingerprinting by ^1^H HR-MAS NMR

*C. gongonha* Mart. is a Brazilian well-known plant, but little attention is given to its molecular profile, except for some usual statements (http://floradigital.ufsc.br). A high-throughput chemical insight of this plant can importantly aid drugs and medicinal chemistry. Thus, an untargeted metabolomic fingerprint profile was acquired for the relevant plant species. Fingerprinting is a qualitative approach, assessing metabolites without needing earlier knowledge and quantification or structure elucidation of the compounds in the sample. In turn, as the given plant lacks chemical data, so carrying out the later step was highly compulsory as given.

**Figure 1:**
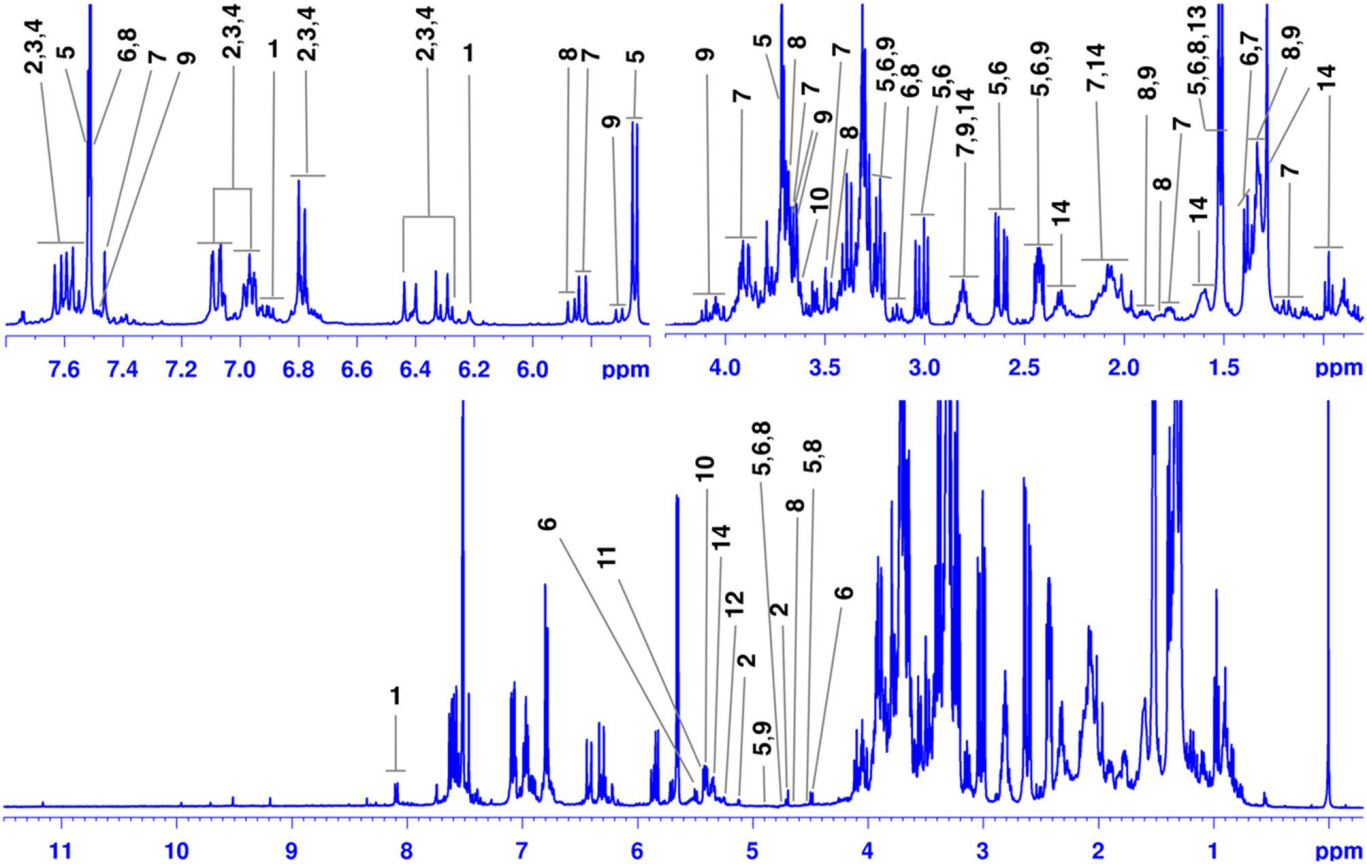
^1^H HR-MAS NMR (400.13 MHz) spectrum showing signal assignments for the metabolites detected in leaf tissue (10 ± 0.05 mg swollen in 40 µL CD_3_OD) from *Citronella gonogonha*. Metabolites: **1**, kaempferol-3-*O*-dihexoside; **2**, caffeoyl glucoside; **3**, 3-*O*-caffeoylquinic acid; **4**, 5-*O*-caffeoylquinic acid; **5**, kingiside; **6**, 8-epi-kingisidic acid; **7**, (7*α*)-7-*O*-methylmorroniside; **8**, (7*β*)-7-*O*-methylmorroniside; **9**, alpigenoside; **10**, sucrose; **11**–**12,** *α*-glucoses; **13**, alanine; **14**, fatty (linolenic) acid. NMR spectrum was produced in TopSpin v3.6.3 software package (Bruker BioSpin: https://www.bruker.com), signal annotations were manually generated in Microsoft PowerPoint v16.56 (https://officecdnmac.microsoft.com) and final figure was generated in GIMP v2.10.24 software package (https://www.gimp.org).

Henceforward, instead molecular extraction, the metabolomics fingerprinting by ^1^H HR-MAS NMR (Fig. 1) was performed for intact leaf tissue of the given plant. In consideration, foremost to the MAS NMR analyses, a gel-like state of the sample with enhanced molecular mobility was essentially realized through a locking solvent (40 µL, CD_3_OD). The anisotropic atomic interactions that hinder spectral resolution, were surpassed by providing a spinning speed of 5000 Hz to the sample at a *so-called* magic angle “θ = 54.74°”. Water resonance that obstructs signals of the metabolites of interest in the samples was circumvented by means of *zgcppr* pulse sequence (Bruker library).

**Figure 2:**
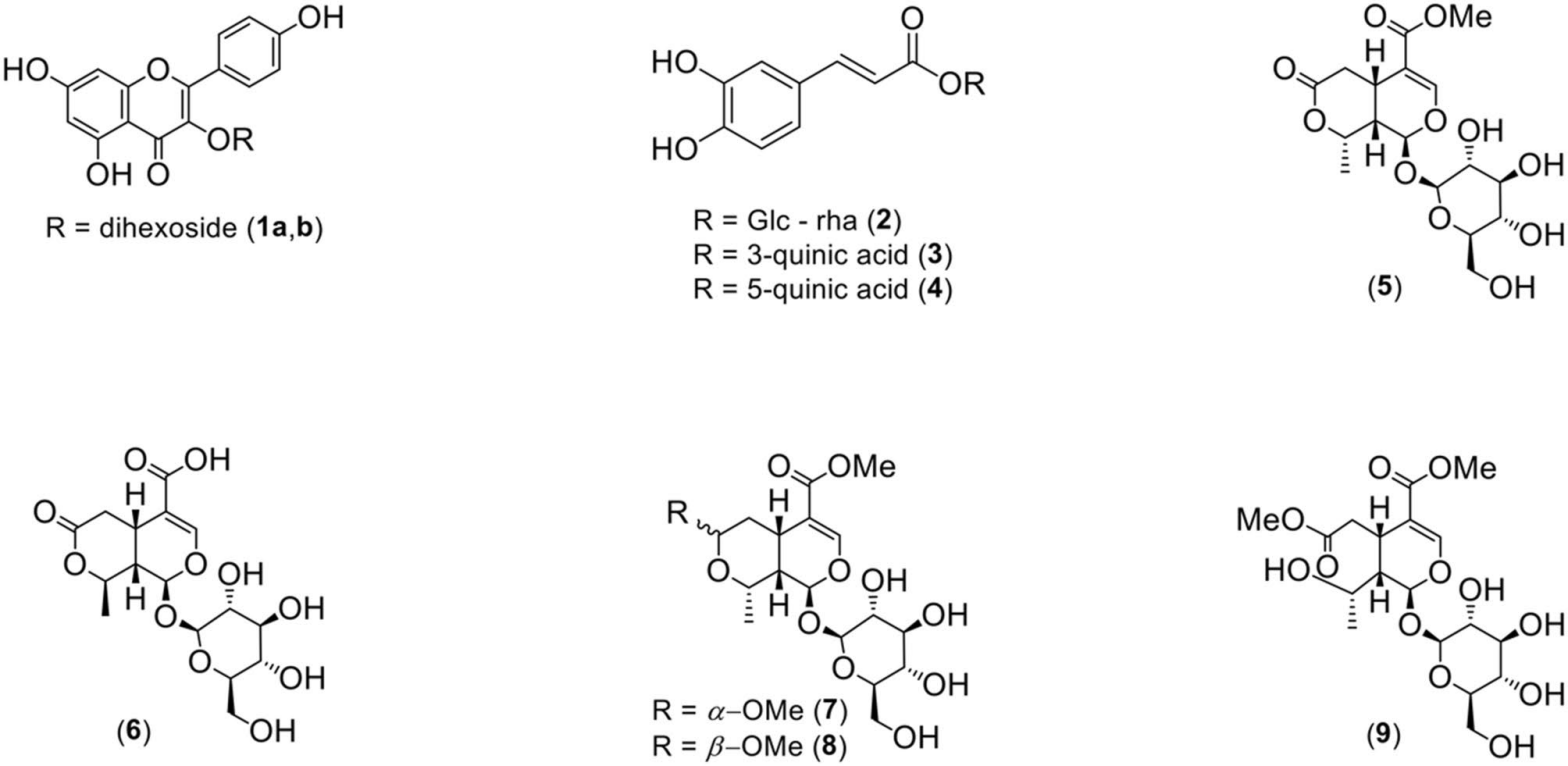
An overview of major metabolites explored in the leaf tissue of *Citronella gongonha* Mart*.* (Cardiopteridaceae). Metabolites: **1a, b**, kaempferol-3-*O*-dihexoside; **2**, caffeoyl glucoside; **3**, 3-*O*-caffeoylquinic acid; **4**, 5-*O*-caffeoylquinic acid; **5**, kingiside; **6**, 8-epi-kingisidic acid; **7**, (7*α*)-7-*O*-methylmorroniside; **8**, (7*β*)-7-*O*-methylmorroniside; **9**, alpigenoside. Chemical structures were produced in ChemDraw Ultra v12.0.2.1076 software package (https://www.cambridgesoft.com).

The established approach allowed acquisition of high-resolution data comparable to that established by the solution-state NMR (Supplementary Fig. S1). This study based on ^1^H HR-MAS NMR (Fig. 1) explored multiple derivatives of the flavonoid, polyphenolics and monoterpenoids as principal compounds, and several other metabolites (Table 2). Major compounds were moreover explored by means of LC–MS/MS tool. In general, such compounds included kaempferol-3-*O*-dihexoside (**1**), caffeoyl glucoside (**2**), 3-*O*-caffeoylquinic acid (**3**), 5-*O*-caffeoylquinic acid (**4**), kingiside (**5**), 8-epi-kingisidic acid (**6**), (7*α*)-7-*O*-methylmorroniside (**7**), (7*β*)-7-*O*-methylmorroniside (**8**) and alpigenoside (**9**) (Fig. 2) along with commonly occurring sucrose (**10**), *α*-glucoses (**11**,**12**), alanine (**13**), and fatty (linolenic) acid (**14**) (Table 2).

Analysis of the ^1^H HR-MAS NMR spectrum (Fig. 1) exhibited many signals with improved relative intensity, in particular from metabolites **2**–**9**. Intensity of the NMR peak inherently depicts quantitative nature of compounds. Therefore, peak relative intensity can show a high medicinal relevance of this plant that produces, especially metabolites **2**–**9** in abundant quantities. In this way, structural clarification of these metabolites is complementary, which beyond the 2D NMR (Supplementary Figs. S8 to S10) was completed by LC–MS/MS (Supplementary Figs. S2 to S7, and Table 1) with the support of literature^3,17,24–28^. To this context, the structural details for major metabolites (**1**–**9**) are summarized and given (Table 2, and Supplementary Information).

**Table 1:** Liquid chromatography coupled to mass spectrometry (LC–MS/MS) analysis of aqueous ethanolic extract of leaf from *Citronella gongonha* Mart. Howard (Cardiopteridaceae).

Metabolites	Mode	Molecular ion (*m*/*z*)	CE (V)	MS^2^ (*m*/*z*)
**1 (a, b)**	MS^2^/ESI^−^	609 [M − H]^−^	40	227, 255, 284
**2**	MS^2^/ESI^−^/ESI^+^	487 [M − H]^−^	10–60	NA
		489 [M + H]^+^		
**3**	MS^2^/ESI^−^	353 [M − H]^−^	22	135, 179, 191
**4**				191
**5 (a)**	MS^2^/ESI^+^	405 [M + H]^+^	10	165, 183, 193, 211, 243, 369, 387
**5 (b)**				165, 193, 211, 243
**6**	MS^2^/ESI^−^/ESI^+^	389 [M − H]^−^	10–60	NA
		391 [M + H]^+^		
**7**	SIR/ESI^+^/ESI^−^	465 [M + HCOO]^−^	10–60	Fragmentation not observed
**8**		443 [M + Na]^+^		Fragmentation not observed
**9**	MS^2^/ESI^−^/ESI^+^	435 [M − H]	10–60	NA
		437 [M + H]^+^		

Metabolites: **1a, b**, kaempferol-3-*O*-dihexoside; **2**, caffeoyl glucoside; **3**, 3-*O*-caffeoylquinic acid; **4**, 5-*O*-caffeoylquinic acid (confirmed with authentic standard compound); **5**(**a**, **b**), kingiside epimers; **6**, 8-epi-kingisidic acid; **7**, (7*α*)-7-*O*-methylmorroniside; **8**, (7*β*)-7-*O*-methylmorroniside; **9**, alpigenoside. MS^2^, mass fragmentation; SIR, single ion reaction; ESI^+^, electrospray ionization in positive mode; ESI^−^, electrospray ionization in negative mode. NA: Data not conclusive due to the lack of standard or MS/MS information in literature.

**Table 2:** Metabolites detected in intact leaf tissue of *Citronella gongonha* Mart. Howard (Cardiopteridaceae) by proton high-resolution magic angle spinning (^1^H HR-MAS) nuclear magnetic resonance (NMR, ^1^H 400.13 MHz; CD_3_OD as a magnetic field locking solvent), and structural elucidation by two-dimensional (2D) heteronuclear (^1^H-^13^C) single quantum correlation (HSQC), long-range heteronuclear (^1^H-^13^C) multiple bond correlation (^L-R^*J*_H-C_ HMBC) and double quantum filter homonuclear (^1^H-^1^H) correlation spectroscopy (DQF-COSY) NMR (^1^H 400.13-^13^C 100.6 MHz; CD_3_OD).

Metabolites	^1^H Chemical shift (ppm), multiplicity, *J* (Hz)	^13^C Chemical shift (ppm)
1	6.21 (d = 2.1, H-6), 6.39 (d = 2.1, H-8), 8.08 (d = 9.0, H-2',6'), 6.90 (m, H-3',5')	99.8 (C-6), 94.5 (C-8), 123.0 (C-1'), 132.0 (C-2',6'), 161.6 (C-4'), 115.0 (C-3',5')
2	7.08 (d = 2.0, H-2), 6.77 (d = 8.2, H-5), 6.95 (dd = 8.2; 2.0, H-6), 7.61 (d = 15.9, H-7), 6.41 (d = 15.9, H-8), 4.70 (d = 7.9, H-1'), 5.11 (d = 3.7, H-1'')	128.1 (C-1), 115.1 (C-2), 149.0 (C-3), 147.0 (C-4), 116.4 (C-5), 123.0 (C-6), 146.8 (C-7), 115.5 (C-8), 169.0 (C-9), 99.9 (C-1'), 94.0 (C-1'')
3,4	7.06 (d = 2.0, H-2) in **3**/7.04 (d = 2.0, H-2) in **4**, 6.78 (d = 8.2, H-5), 6.96 (dd = 8.2; 2.0, H-6), 7.59 (d = 15.9, H-7) in **3**/7.55 (d = 15.9, H-7) in **4**, 6.30 (d = 15.9, H-8) in **3**/6.27 (d = 15.9, H-8) in **4**	127.8 (C-1), 115.1 (C-2), 147.0 (C-3), 149.5 (C-4), 116.4 (C-5), 122.9 (C-6), 146.8 (C-7), 115.1 (C-8), 169.0 (C-19)
5	5.65 (d = 6.1, H-1), 7.52 (s, H-3), 3.22 (m, H-5), 3.01 (dd = 17.1; 7.6, H-6a), 2.61 (17.1; 6.0, H-6b), 4.76 (m, H-8), 2.42 (dddd = 13.6; 10.2; 6.1; 4.1, H-9), 3.71 (s, H-10), 4.87 (br, d, H-1')	94.4 (C-1), 154.1 (C-3), 111.6 (C-4), 28.0 (C-5), 34.4 (C-6), 174.5 (C-7), 76.5 (C-8), 40.0 (C-9), 19.0 (C-10), 168.1 (C-11), 51.5 (C-12), 100.0 (C-1')
6	5.49 (d = 7.7, H-1), 7.51 (s, H-3), 3.11 (m, H-5), 3.01 (dd = 17.1; 7.6, H-6a), 2.61 (17.1; 6.0, H-6b), 4.76 (m, H-8), 2.42 (dddd = 13.6; 10.2; 6.1; 4.1, H-9), 1.52 (d = 6.8, H-10), 4.48 (d = 7.8, H-1')	96.1 (C-1), 154.1 (C-3), 111.6 (C-4), 28.0 (C-5), 34.4 (C-6), 174.5 (C-7), 76.5 (C-8), 40.0 (C-9), 19.0 (C-10), 168.2 (C-11), 98.1 (C-1')
7	5.83 (d = 9.3, H-1), 7.48 (s, H-3), 2.80 (m, H-5), 1.18 (dt = 13.0; 10.0, H-6a), 2.07 (m, H-6b), 3.50 (s, 7-O-CH_3_), 3.91 (m, H-8), 1.80 (m, H-9), 1.39 (d = 6.9, H-10), 3.69 (s, 11-O-CH_3_), 4.87 (br, d, H-1')	95.5 (C-1), 153.5 (C-3), 111.6 (C-4), 31.6 (C-5), 37.4 (C-6), 73.3 (C-8), 40.0 (C-9), 19.7 (C-18), 168.1 (C-11), 51.6 (11-O-CH_3_), 100.0 (C-1')
8	5.87 (d = 9.2, H-1), 7.51 (s, H-3), 3.11 (m, H-5), 1.52 (d = 6.8, H-6a), 1.92 (m, H-6b), 4.76 (m, H-7), 3.37 (s, 7-O-CH_3_), 4.55 (m, H-8), 1.82 (m, H-9), 1.35 (d = 6.9, H-10), 3.70 (s, 11-O-CH_3_), 4.65 (d = 7.8, H-1')	95.5 (C-1), 153.7 (C-3), 111.6 (C-4), 28.1 (C-5), 34.3 (C-6), 99.9 (C-7), 51.5 (7-O-CH_3_), 65.6 (C-8), 40.3 (C-9), 22.2 (C-10), 168.1 (C-11), 51.6 (11-O-CH_3_), 99.8 (C-1')
9	5.71 (d = 8.5, H-1), 7.48 (br, s, H-3), 3.23 (m, H-5), 2.42 (dddd = 13.6; 10.2; 6.1; 4.1, H-6a), 2.81 (m, H-6b), 3.67 (s, 7-O-CH_3_), 4.04 (m, H-8), 1.90 (m, H-9), 1.35 (d = 6.7, H-10), 3.69 (s, 11-O-CH_3_), 4.87 (m, H-1')	97.6 (C-1), 153.4 (C-3), 111.6 (C-4), 31.6 (C-5), 37.8 (C-6), 175.0 (C-7), 51.5 (7-O-CH_3_), 68.0 (C-8), 46.0 (C-9), 22.2 (C-10), 168.8 (C-11), 51.5 (11-O-CH_3_), 100.0 (C-1')
10	5.39 (d = 3.7, H-1), 3.44 (m, H-2), 3.64 (m, H-1')	93.3 (C-1), 73.1 (C-2), 63.5 (C-1'), 105.4 (C-2')
11, 12	5.42 (d = 3.7, H-1) in **11**, 5.24 (d = 3.7, H-1) in **12**	93.0 (C-1) in **11**, 92.1 (C-1) in **12**
13	1.52 (d = 6.8, H-3)	–
14	2.32 (m, H-2), 1.60 (m, H-3), 1.28 (m H-4 to H-7), 2.06 (m, H-8,17), 5.34 (m), 2.81 (m, H-11,14), 0.97 (t = 7.7, H-18),	174.7 (C-1), 35.0 (C-2), 26.0 (C-3), 30.5 (C-4 to C-7), 28.0 (C-8,17), 128.9 (-C = C-), 26.5 (C-11, 14), 14.5 (CH_3_-18)

Multiplicity: brd, broad doublet; brs, broad singlet; d, doublet; dd, doublet of doublets; dddd, doublet of doublet of doublet of doublets; dt, doublet of triplet; m, multiplet; s, singlet. Metabolites: **1**, kaempferol-3-*O*-dihexoside; **2**, caffeoyl glucoside; **3**, 3-*O*-caffeoylquinic acid; **4**, 5-*O*-caffeoylquinic acid; **5**, kingiside; **6**, 8-epi-kingisidic acid; **7**, (7*α*)-7-*O*-methylmorroniside; **8**, (7*β*)-7-*O*-methylmorroniside; **9**, alpigenoside.

### Structural elucidation of metabolites (1–9)

Chemical compounds (**1**–**9**, and in Table 2) described in this work were investigated in complex mixture of the leaf tissue from *C. gongonha* Mart. As a principal tool, the ^1^H HR-MAS and 2D NMR was supplemented by the LC–MS/MS analysis elucidating the following compounds.

**Figure 3:**
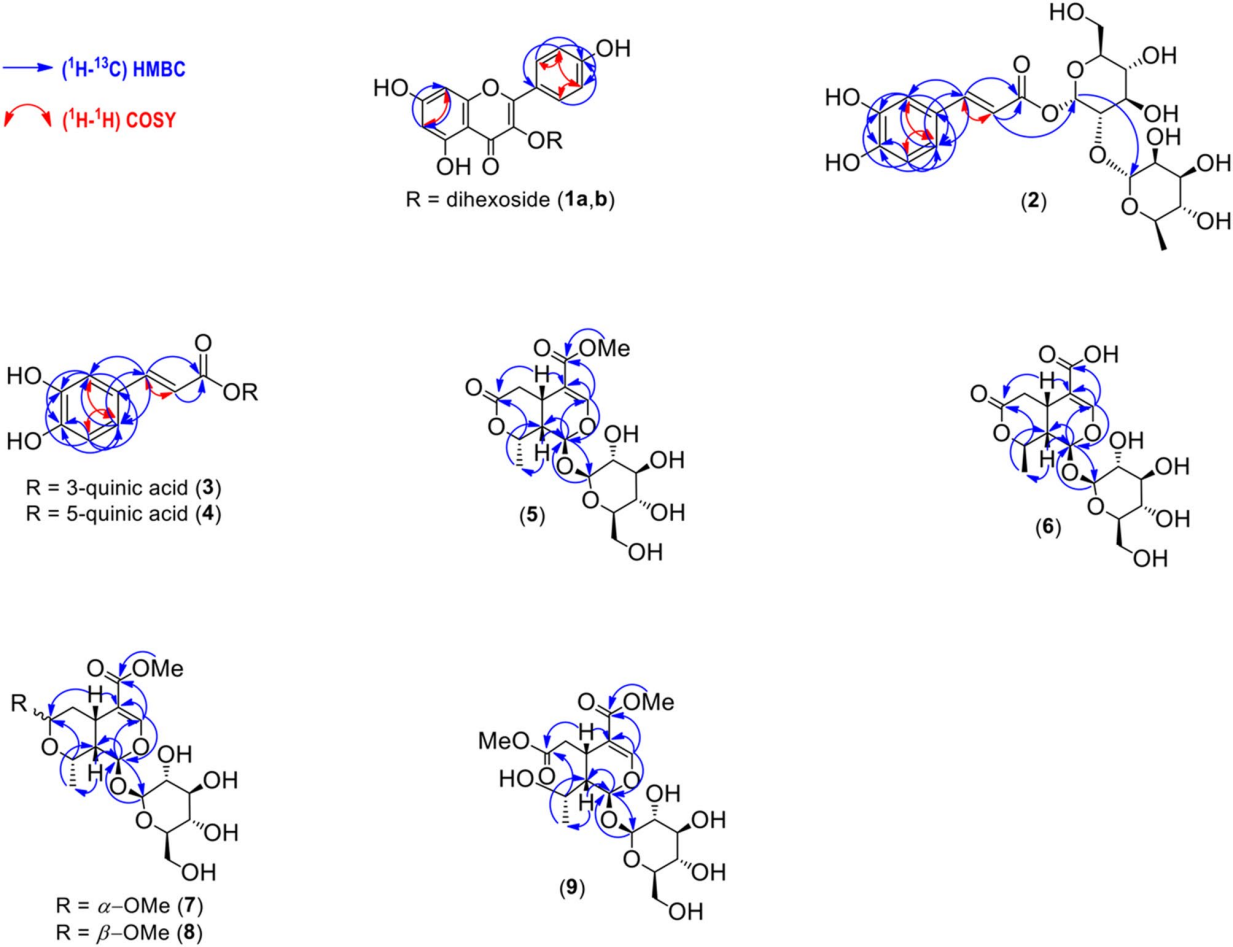
2D NMR correlations for the major metabolites (**1**–**9**) explored in leaf tissue of *Citronella gongonha* Mart. Metabolites: **1a, b**, kaempferol-3-*O*-dihexoside; **2**, caffeoyl glucoside; **3**, 3-*O*-caffeoylquinic acid; **4**, 5-*O*-caffeoylquinic acid; **5**, kingiside; **6**, 8-epi-kingisidic acid; **7**, (7*α*)-7-*O*-methylmorroniside; **8**, (7*β*)-7-*O*-methylmorroniside; **9**, alpigenoside. All of the chemical structures were produced in ChemDraw Ultra v12.0.2.1076 software package (https://www.cambridgesoft.com).

Compound (**1**) was distinguished by LC–MS/MS under the electrospray ionization in negative mode (ESI^−^) as derivatives of kaempferol-3-*O*-dihexoside (**1a**, **b**). Spectrometric analyses for these derivatives indicated one molecular ion peak at *m/z* 609. Using the same collision energy (40 V), the MS^2^ fragmentation for relevant compounds provided the transitions of *m/z* 227, 255 and 284 (Supplementary Figs. S2 to S3, Table 1), which followed the same fragmentation pattern as described previously by He et al.^26^. Conversely, in NMR data due to lower peaks intensity, it was cumbersome to fully determine a complete structure for the given compound **1** (**a**, **b**). Compound **1** is a biologically active metabolite from the flavonoid family, and widely distributed in nature, including plants^3,8,9,29^. Conforming the ^1^H HR-MAS NMR data (Table 2), metabolite **1** showed six methines protons of H-2′/6′ (δ_H_ 8.08, d, *J* = 9.0 Hz), H-3′/5′ (δ_H_ 6.90, m), H-6 (δ_H_ 6.21, d, *J* = 2.1 Hz) and of H-8 (δ_H_ 6.39, d, *J* = 2.1 Hz), suggesting two aromatic rings from basic skeleton (B and A) of flavonoid**.** The ^13^C data for **1** were completed in the multiplicity edited 2D (^1^H-^13^C) HSQC NMR (Supplementary Fig. S8), demonstrating carbons at δ_C_ 132.0 (C-2′/C-6′), 115.0 (C-3′/C-5′), 99.8 (C-6) and 94.5 (C-8). The analysis of 2D (^1^H-^13^C) HMBC NMR (Supplementary Fig. S9, and Fig. 3) confirmed the presence of three rings by providing key correlations from H-2′/H-6′ (δ_H_ 8.08, d, *J* = 9.0 Hz) to C-4′ (δ_C_ 161.6), from H-3′/H-5′ (δ_H_ 6.90, m) to C-1′ (δ_C_ 123.0) and C-5′/C-3′ (δ_C_ 115.0), from H-6 (δ_H_ 6.21, d, *J* = 2.1 Hz) to C-8 (δ_C_ 94.5) and from H-8 (δ_H_ 6.39, d, *J* = 2.1 Hz) to C-6 (δ_C_ 99.9). The LC–MS/MS analysis indicated **1** has two derivatives (**1a**, **b**)^26^ while the NMR peak attributions (Table 2) were highly low in intensity but identical to those in literature^3^.

Polyphenolic compounds, such as commonly occurring derivatives of hydroxycinnamic acid, also known as caffeic acid or additional analogues from this class represent important biological functions and applications^10,11^. In such a class of compounds, this work explored the following derivatives.

Mass spectrometry analyses were performed in both modes (ESI^−^ and ESI^+^), but due to lack of proper standard compound or data availability in literature, this compound was difficult to recognize. Nevertheless, the NMR analysis revealed this as a derivative of caffeoyl glucoside (**2**). Similarly, ^1^H HR-MAS NMR (Table 2) established metabolite **2** based on the following signals from aromatic ring protons of H-2 (δ_H_ 7.08, d, *J* = 2.0 Hz), H-5 (δ_H_ 6.77, d, *J* = 8.2 Hz) and H-6 (δ_H_ 6.95, dd, *J* = 8.2; 2.0 Hz), and the vinylic protons of H-7 (δ_H_ 7.61, d, *J* = 16.0 Hz) and H-8 (δ_H_ 6.41, d, *J* = 16.0 Hz), while the attached glucose units were illustrated by the anomeric protons of *β*-H-1′ (δ_H_ 4.70, d, *J* = 7.9 Hz) and *α*-H-1″ (δ_H_ 5.11, d, *J* = 3.7 Hz). The carbon assignments of **2** were completed in multiplicity edited 2D (^1^H-^13^C) HSQC NMR (Supplementary Fig. S8) that showed carbons at δ_C_ 115.1 (C-2), 116.4 (C-5), 123.0 (C-6), 146.8 (C-7), 115.4 (C-8), 99.9 (C-1′) and 94.0 (C-1″). Analysis of 2D (^1^H-^13^C) HMBC NMR (Supplementary Fig. S9, and Fig. 3) confirmed **2** by means of basic correlations from H-2 (δ_H_ 7.08, d, *J* = 2.0 Hz) to C-3 (δ_C_ 149.0), C-4 (δ_C_ 147.0) and C-6 (δ_C_ 123.0), from H-5 (δ_H_ 6.77, d, *J* = 8.2 Hz) to C-1 (δ_C_ 128.1), C-3 (δ_C_ 149.0), C-4 (δ_C_ 147.0) and C-6 (δ_C_ 123.0), from H-6 (δ_H_ 6.95, dd, *J* = 8.2; 2.0 Hz) to C-2 (δ_C_ 115.1), C-3 (δ_C_ 149.0) and C-4 (δ_C_ 147.0), from H-7 (δ_H_ 7.61, d, *J* = 16.0 Hz) to C-1 (δ_C_ 123.0), C-2 (δ_C_ 115.1) and C-9 (δ_C_ 169.0), from H-8 (δ_H_ 6.41, d, *J* = 16.0 Hz) to C-1 (δ_C_ 128.1), C-9 (δ_C_ 169.0) and C-1′ (δ_C_ 99.9), from H-1′ (δ_H_ 4.70, d, *J* = 7.9 Hz) to C-1″ (94.0), 77.7 and 75.1, from H-1″ (δ_H_ 5.11, d, *J* = 3.7 Hz) to C-1′ (99.9) representing the attachment of two glucose units. Without additional correlations, the chemical structure of **2** was completed as a caffeoyl moiety attached to *β*-D-glucose and *α*-D-rhamnose (Table 2), following literature^17^.

In line with literature^27^, sample analysis by LC–MS/MS in ESI^−^ mode revealed the presence of two caffeoylquinic acids—e.g., 3-*O*-caffeoylquinic acid (**3**) and 5-*O*-caffeoylquinic acid (**4**). These derivatives were differentiated by giving single molecular ion peak at *m/z* 353. However, using a similar collision energy (22 V), MS^2^ fragments for compound **3** were *m/z* 135, 179 and 191, when compared with **4** that exhibited a majority of *m/z* 191 (Supplementary Figs. S4 to S5, and Table 1) that was similar to the previously published results^27^. In ^1^H HR-MAS NMR (Table 2), metabolites **3** and **4** were observed identical to **2**, but were different due to the peaks from the aromatic ring protons and the vinylic protons. In this regard, ^1^H HR-MAS NMR presented the aromatic ring protons of H-2 (δ_H_ 7.06, d, *J* = 2.0 Hz), H-5 (δ_H_ 6.78, d, *J* = 8.2 Hz) and H-6 (δ_H_ 6.96, dd, *J* = 8.2; 2.0 Hz), and the vinylic protons of H-7 (δ_H_ 7.59, d, *J* = 16.0 Hz) and H-8 (δ_H_ 6.30, d, *J* = 16.0 Hz) in **3**, and except for the protons of H-2 (δ_H_ 7.04, d, *J* = 2.0 Hz), H-7 (δ_H_ 7.55, d, *J* = 16.0 Hz) and H-8 (δ_H_ 6.27, d, *J* = 16.0 Hz), the remaining chemical shifts from **4** were compatible with those observed for metabolite **3**. Carbon assignments in **3, 4** were accomplished by 2D (^1^H-^13^C) HSQC NMR (Supplementary Fig. S8), showing carbons at δ_C_ 115.1 (C-2), 116.4 (C-5), 123.0 (C-6), 146.8 (C-7), 115.1 (C-8) and 169.0 (C-9). In 2D (^1^H-^13^C) HMBC NMR (Supplementary Fig. S9, and Fig. 3), **3**, **4** given key correlations from H-2 (δ_H_ 7.06, d, *J* = 2.0 Hz) to C-3 (δ_C_ 147.0), C-4 (δ_C_ 149.5) and C-6 (δ_C_ 123.0), from H-5 (δ_H_ 6.78, d, *J* = 8.2 Hz) to C-1 (δ_C_ 127.8), C-3 (δ_C_ 147.0), C-4 (δ_C_ 149.5) and C-6 (δ_C_ 123.0), from H-6 (δ_H_ 6.96, dd, *J* = 8.2; 2.0 Hz) to C-2 (δ_C_ 115.1), C-3 (δ_C_ 147.0) and C-4 (δ_C_ 149.5), from H-7 (δ_H_ 7.61, d, *J* = 16.0 Hz) to C-2 (δ_C_ 115.1), C-6 (δ_C_ 123.0) and C-9 (δ_C_ 169.0), from H-8 (δ_H_ 6.27, d, *J* = 16.0 Hz) to C-1 (δ_C_ 127.1) and C-9 (δ_C_ 169.0). When compared with the MS/MS result, the 2D NMR correlations did not correspond the presence of quinic acid attachment to the caffeoyl segments in **3** or **4** (Table 2).

Moreover, the iridoids or secoiridoids are part of naturally occurring monoterpenoids group of compounds that can be major taxonomic markers of plants, especially *C. gongonha* Mart. In such a class of compounds, this work determined different derivatives such as kingiside (**5**), 8-epikingisidic acid (**6**), (7*α*)-7-*O*-methylmorroniside (**7**), (7*β*)-7-*O*-methylmorroniside (**8**) and alpigenoside (**9**) respectively.

Kingiside (**5**) was characterized by LC–MS/MS analysis in the ESI^+^, representing two molecular ion peaks of *m/z* 405, indicating probably two epimers (**a**, **b**) of **5**. By providing a 10 V collision energy, **5a** showed the MS^2^ fragments of *m/z* 165, 183, 193, 211, 243, 369 and 387. Although, **5b** given the following MS^2^ fragments of *m/z* 165, 193, 211 and 243 suggesting the presence of two epimers of **5**^28^ (Supplementary Figs. S6–S7, and Table 1). In order to analyzed ^1^H HR-MAS NMR data (Fig. 1 and Table 2), metabolite **5** was confirmed on the basis of unique peak of H-1 (δ_H_ 5.65, d, *J* = 6.1 Hz). This metabolite was moreover characterized through the protons of H-1 (δ_H_ 5.65, d, *J* = 6.1 Hz), H-3 (δ_H_ 7.51, s), H-5 (δ_H_ 3.22, m), H-6a/H-6b [(δ_H_ 3.01, dd, *J* = 17.1; 7.6 Hz)/(δ_H_ 2.61, dd, *J* = 17.1; 6.0 Hz)], H-8 (δ_H_ 4.76 dd *J* = 6.6; 4.0), H-9 (δ_H_ 2.42, dddd, *J* = 13.6; 10.2; 6.1; 4.1), the methyl protons of H-10 (δ_H_ 1.52, d, *J* = 6.8 Hz), methoxy protons of H-12 (δ_H_ 3.71, s), and the glucose proton of H-1′ (δ_H_ 4.87, br d). The 2D (^1^H-^13^C) HSQC NMR (Supplementary Fig. S8) in related metabolite displayed the carbons at C-1 (δ_C_ 94.4), C-3 (δ_C_ 154.1), C-5 (δ_C_ 28.0), C-6 (δ_C_ 34.0), C-8 (δ_C_ 76.5), C-9 (δ_C_ 40.0), C-10 (δ_C_ 19.3), C-12 (δ_C_ 51.5) and C-1′ (δ_C_ 100.0). The main correlations in these compounds through the 2D (^1^H-^13^C) HMBC NMR (Supplementary Fig. S9, and Fig. 3) were from H-1 (δ_H_ 5.65, d, *J* = 6.1 Hz) to C-5 (δ_C_ 28.0), C-8 (δ_C_ 76.5) and C-1′ (δ_C_ 100.0), from H-3 (δ_H_ 7.51, s) to C-1 (δ_C_ 94.4), C-4 (δ_C_ 111.6), C-5 (δ_C_ 28.0) and C-11 (δ_C_ 168.1), from H-5 (δ_H_ 3.22, m) to C-11 (δ_C_ 154.1), C-4 (δ_C_ 111.6), C-7 (δ_C_ 174.5), C-8 (δ_C_ 76.5) and C-9 (δ_C_ 40.0), from H-6a/H-6b [(δ_H_ 3.01, dd, *J* = 17.1; 7.6 Hz)/(δ_H_ 2.61, dd, *J* = 17.1; 6.0 Hz)] to C-4 (δ_C_ 111.6), C-5 (δ_C_ 28.0), C-7 (δ_C_ 174.5) and C-9 (δ_C_ 40.0), from H-8 (δ_H_ 4.76, m) to only C-10 (δ_C_ 19.0), from H-9 (δ_H_ 2.42, dddd, *J* = 13.6; 10.2; 6.1; 4.1) to C-1 (δ_C_ 94.4), C-4 (δ_C_ 111.6), C-5 (δ_C_ 28.0) and C-7 (δ_C_ 174.5), from methyl protons of H-10 (δ_H_ 1.52, d, *J* = 6.8 Hz) to C-7 (δ_C_ 174.5), C-8 (δ_C_ 76.5) and C-9 (δ_C_ 40.0), from the methoxy protons of H-12 (δ_H_ 3.71, s) to C-11 (δ_C_ 168.1) as well as from glucose proton of H-1′ (δ_H_ 4.87, br d) to C-1 (δ_C_ 94.4), 77.8 and 75.2 (Supplementary Fig. S9). These results were in agreement with the literature that isolated **5** from *Lonicera alpigena* (Caprifoliaceae)^25^ and *Gentiana rhodantha* (Gentianaceae)^30^, yet, **5** in this work was directly detected in the mixture sample.

Similar to the analyses for compound **2**, metabolite **6** was consistently not detected due to the lack of MS^2^ fragmentation data in literature but were observed in NMR. ^1^H HR-MAS NMR (Table 2) determined metabolite **6** based on the pyran ring protons of H-1 (δ_H_ 5.49, d, *J* = 7.7 Hz) and H-3 (δ_H_ 7.51, s), while H-5 (δ_H_ 3.11, m), H-6a/H-6b [(δ_H_ 3.01, dd, *J* = 17.1; 7.6 Hz)/(δ_H_ 2.61, dd, *J* = 17.1; 6.0 Hz)], H-8 (δ_H_ 4.76 dd *J* = 6.6; 4.0), H-9 (δ_H_ 2.42, dddd, *J* = 13.6; 10.2; 6.1; 4.1), methyl protons of H-10 (δ_H_ 1.52, d, *J* = 6.8 Hz) and a *β*-glucose proton of H-1′ (δ_H_ 4.48 d *J* = 7.8 Hz). All carbons in **6** were explored by 2D (^1^H-^13^C) HSQC NMR (Supplementary Fig. S8) at C-1 (δ_C_ 96.1), C-3 (δ_C_ 154.1), C-5 (δ_C_ 28.0), C-6 (δ_C_ 34.4), C-8 (δ_C_ 76.5), C-9 (δ_C_ 40.0), C-10 (δ_C_ 19.0) and C-1′ (δ_C_ 98.1). The multiple bond proton to carbon correlations by 2D (^1^H-^13^C) HMBC NMR (Supplementary Fig. S9, and Fig. 3) were observed from H-1 (δ_H_ 5.49, d, *J* = 7.7 Hz) to C-1′ (δ_C_ 98.1), from H-3 (δ_H_ 7.51, s) to C-1 (δ_C_ 96.1), C-4 (δ_C_ 111.6), C-5 (δ_C_ 28.0) and C-11 (δ_C_ 168.2), from H-5 (δ_H_ 3.11, m) to C-1 (δ_C_ 96.1) and C-4 (δ_C_ 111.6), from H-6a/H-6b [(δ_H_ 3.01, dd, *J* = 17.1; 7.6 Hz)/(δ_H_ 2.61, dd, *J* = 17.1; 6.0 Hz)] to C-4 (δ_C_ 111.6), C-5 (δ_C_ 28.0), C-7 (δ_C_ 174.5) and C-9 (δ_C_ 40.0), from H-8 (δ_H_ 4.76, dd *J* = 6.6; 4.0 Hz) to C-10 (δ_C_ 19.0) and from H-9 (δ_H_ 2.42, dddd, *J* = 13.6; 10.2; 6.1; 4.1 Hz) to C-7 (δ_C_ 174.5). The 2D (^1^H-^1^H) COSY NMR (Supplementary Fig. S10, and Fig. 3) supported mutual correlations from proton of H-5 (δ_H_ 3.11, m) with H-6a/H-6b [(δ_H_ 3.01, dd, *J* = 17.1; 7.6 Hz)/(δ_H_ 2.61, dd, *J* = 17.1; 6.0 Hz)] and H-9 (δ_H_ 2.42, dddd, *J* = 13.6; 10.2; 6.1; 4.1 Hz). All structural details were agreeing the previously published results^25,30^.

In addition to the abovementioned metabolites, other derivatives of monoterpenoids or the secoiridoids explored in the given plant incorporated (7*α*)-7-*O*-methylmorroniside (**7**), (7*β*)-7-*O*-methylmorroniside (**8**) and alpigenoside (**9**). The experimental results were in agreement with the published data^24^ that shown the isolation and characterization for related metabolites in pitcher plant. With little differences in the NMR chemical shifts, metabolites **7**, **8**, and **9** were distinguished by the pyran ring proton such as H-1 (δ_H_ 5.83, d, *J* = 9.3 Hz) in **7**, H-1 (δ_H_ 5.87, d, *J* = 9.3 Hz) in **8** and H-1 (δ_H_ 5.71, d *J* = 8.5 Hz) in **9**.

The data obtained by LC–MS/MS for **7** and **8** were not conclusive. Two individual peaks were obtained through a single ion reaction (SIR) in the positive (ESI^+^) and negative (ESI^−^) modes. Consistently, formate (CHOO^−^) and sodium (Na^+^) adducts for compounds **7** and **8** were identified as 465 ([*M* + CHOO^−^]^−^) and 443 ([*M* + Na^+^]), as reported in the literature for the given compounds^24^. MS^2^ analysis for these adducts with the collision energies ranging from 10 to 60 V did not produce fragmentation pattern with *m*/*z* above 100. Additionally, ^1^H HR-MAS NMR (Table 2) revealed (7*α*)-7-*O*-methylmorroniside (**7**)^24^ due to the pyran ring protons of H-1 (δ_H_ 5.83, d, *J* = 9.3 Hz) and H-3 (δ_H_ 7.48 br, s), and H-5 (δ_H_ 2.80, m), H-6a/H-6b [(δ_H_ 1.18, dt, *J* = 13.0; 10.0 Hz)/(δ_H_ 2.07, m)], methoxy protons of H-7 (δ_H_ 3.50, s), H-8 (δ_H_ 3.91, m), H-9 (δ_H_ 1.80, m), methyl protons of H-10 (δ_H_ 1.39, d, *J* = 6.9 Hz), methoxy protons of H-11 (δ_H_ 3.69, s) and the glucose proton of H-1′ (δ_H_ 4.87, m). All carbons in **7** were discriminated by 2D (^1^H-^13^C) HSQC NMR (Supplementary Fig. S8) at C-1 (δ_C_ 95.5), C-3 (δ_C_ 153.5), C-5 (δ_C_ 31.6), C-6 (δ_C_ 37.4), C-8 (δ_C_ 74.0), C-9 (δ_C_ 40.0), C-10 (δ_C_ 19.7), C-11 (δ_C_ 51.6) and C-1′ (δ_C_ 100.0). Moreover, the chemical structure of **7** was completed with the 2D (^1^H-^13^C) HMBC NMR (Supplementary Fig. S9, and Fig. 3). This presented key correlation from H-1 (δ_H_ 5.83, d, *J* = 9.3 Hz) to C-8 (δ_C_ 74.0) and C-1′ (δ_C_ 100.0), from H-3 (δ_H_ 7.48 br, s) to C-4 (δ_C_ 111.6), C-5 (δ_C_ 31.6) and C-11 (δ_C_ 168.2), from H-10 (δ_H_ 1.39, d, *J* = 6.9 Hz) to C-8 (δ_C_ 74.0) and C-9 (δ_C_ 40.0), respectively.

Following ^1^H HR-MAS NMR analysis (Table 2), (7*β*)-7-*O*-methylmorroniside (**8**)^24^ was established based on the protons in pyran ring of H-1 (δ_H_ 5.87, d, *J* = 9.3 Hz) and H-3 (δ_H_ 7.51, s), and H-5 (δ_H_ 3.11, m), methylene protons of H-6a/H-6b [(δ_H_ 1.52, d, *J* = 6.8 Hz)/(δ_H_ 1.92, m)], methine proton of H-7 (δ_H_ 4.76, m), methoxy (-*β-O*-CH_3_) protons of H-7 (δ_H_ 3.37, s), H-8 (δ_H_ 4.55, m), H-9 (δ_H_ 1.82, m), methyl protons of H-10 (δ_H_ 1.35, d *J* = 6.9 Hz), methoxy protons of H-11 (δ_H_ 3.70, s) and the *β*-glucose proton of H-1′ (δ_H_ 4.65, d *J* = 7.8 Hz). Carbon assignments were proven through the 2D (^1^H-^13^C) HSQC NMR (Supplementary Fig. S8) representing C-1 (δ_C_ 95.5), C-3 (δ_C_ 153.7), C-5 (δ_C_ 28.0), C-6 (δ_C_ 34.3), C-7 (δ_C_ 99.9), *β-O*-CH_3_-7 (δ_C_ 51.5), C-8 (δ_C_ 65.6), C-9 (δ_C_ 40.3), C-10 (δ_C_ 22.2), *O*-CH_3_-11 (δ_C_ 51.6) and C-1′ (δ_C_ 99.9). Additionally, the 2D (^1^H-^13^C) HMBC NMR (Supplementary Fig. S9, and Fig. 3) confirmed key correlations in **8** from H-1 (δ_H_ 5.87, d, *J* = 9.3 Hz) to C-1′ (δ_C_ 99.9), from H-3 (δ_H_ 7.51, s) to C-1 (δ_C_ 95.5), C-4 (δ_C_ 111.6), C-5 (δ_C_ 28.0) and C-11 (δ_C_ 168.1), from H-5 (δ_H_ 3.11, m) to C-4 (δ_C_ 111.6) and C-7 (δ_C_ 99.9), from H-6a/H-6b [(δ_H_ 1.52, d, *J* = 6.8 Hz)/(δ_H_ 1.92, m)] to C-9 (δ_C_ 40.3), from H-8 (δ_H_ 4.55, m) to C-7 (δ_C_ 99.9) and from H-11 (δ_H_ 3.70, s) to C-11 (δ_C_168.1). Several protons, in particular H-8 (δ_H_ 4.55, m) in **8** was confirmed in the 2D (^1^H-^1^H) COSY NMR (Supplementary Fig. S10, and Fig. 3).

Consequently, another derivative of alpigenoside (**9**)^24^ was confirmed by NMR rather than spectrometry analysis of the sample that was stored for a long time (four years). Hu et al.^24^ disclosed that the degree of conversion of **9** to **5** is sensitive to subtle differences in drying and storage conditions, consequently **9** can cyclized to **5** along the storage duration. So, ^1^H HR-MAS NMR (Table 2) determined metabolite **9** based on typical signals from the protons in pyran ring of H-1 (δ_H_ 5.71, d *J* = 8.5 Hz) and H-3 (δ_H_ 7.48, br, s), and H-5 (δ_H_ 3.23, m), methylene protons of H-6a/H-6b [(δ_H_ 2.42, dddd *J* = 13.6; 10.2; 6.1; 4.1 Hz)/(δ_H_ 2.81, m)], methoxy (*-O*-CH_3_) protons of H-7 (δ_H_ 3.67, s), H-8 (δ_H_ 4.04, m), H-9 (δ_H_ 1.90, m), methyl protons of H-10 (δ_H_ 1.35, d *J* = 6.9 Hz), methoxy protons of H-11 (δ_H_ 3.69, s) and the glucose proton of H-1′ (δ_H_ 4.87, m). The carbon assignments in **9** were accomplished by 2D (^1^H-^13^C) HSQC NMR (Supplementary Fig. S8) demonstrating C-1 (δ_C_ 97.6), C-3 (δ_C_ 153.4), C-5 (δ_C_ 31.6), C-6 (δ_C_ 37.8), *O*-CH_3_-7 (δ_C_ 51.5), C-8 (δ_C_ 68.0), C-9 (δ_C_ 46.0), C-10 (δ_C_ 22.2), *O*-CH_3_-11 (δ_C_ 51.5) and C-1′ (δ_C_ 100.0). The 2D (^1^H-^13^C) HMBC NMR (Supplementary Fig. S9, and Fig. 3) uncovered the main correlations from H-1 (δ_H_ 5.71, d *J* = 8.5 Hz) to C-1′ (δ_C_ 100.0), from H-3 (δ_H_ 7.48, br s) to C-1 (δ_C_ 97.6), C-4 (δ_C_ 111.6), C-5 (δ_C_ 31.6) and C-11 (δ_C_ 168.8), from H-5 (δ_H_ 3.23, m) to C-7 (δ_C_ 175.0), from H-6a/H-6b [(δ_H_ 2.42, dddd *J* = 13.6; 10.2; 6.1; 4.1 Hz)/(δ_H_ 2.81, m)] to C-4 (δ_C_ 111.6), C-5 (δ_C_ 31.6), C-7 (δ_C_ 175.0) and C-9 (δ_C_ 46.0), from H-9 (δ_H_ 1.90, m) to C-1 (δ_C_ 97.6), C-8 (δ_C_ 68.0) and C-10 (δ_C_ 22.2), from H-10 (δ_H_ 1.35, d *J* = 6.9 Hz) to C-8 (δ_C_ 68.0) and C-9 (δ_C_ 46.0), and from methoxy protons of H-11 (δ_H_ 3.69, s) to the carbonyl C-11 (δ_C_ 168.8). In line with the described metabolites (**1**–**8**), the 2D (^1^H-^1^H) COSY NMR (Supplementary Fig. S10, and Fig. 3) was highly useful for the confirmation of certain positions in individual compounds, comprising a proton of H-8 (δ_H_ 4.55, m) in **9**, respectively.

^1^H HR-MAS NMR is dynamic tool that captures profound details, such as seasonality and environmental influence over plant metabolome^3,17^. To seek this information, the ^1^H HR-MAS NMR fingerprint data of *C. gongonha* Mart. were analyzed in PCA tool.

### Principal component analysis (PCA)

**Figure 4:**
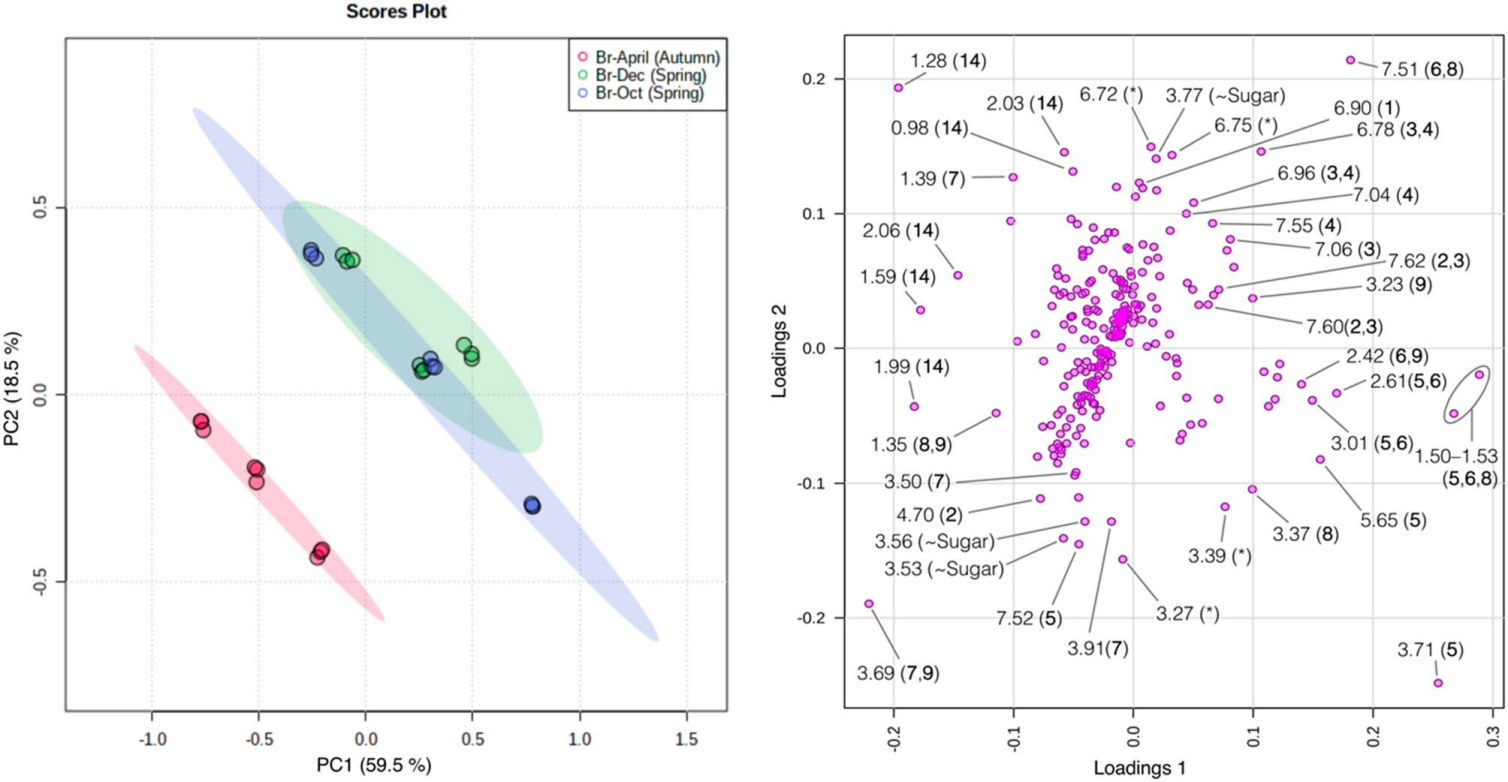
PCA over the ^1^H HR-MAS NMR fingerprints representing seasonal influence for major metabolites. NMR data were processed in TopSpin v3.6.3 software package and preprocessed in analysis of mixture (AMIX) v3.9.12 software package (Bruker BioSpin: https://www.bruker.com), PCA analysis was performed in the MetaboAnalyst v5.0 (https://www.metaboanalyst.ca/). Representations for discriminatory metabolites indicating main variables in the loading plot were manually produced in Microsoft PowerPoint v16.56 (https://officecdnmac.microsoft.com), and the GIMP v2.10.24 software package (https://www.gimp.org) was used for final figure generation. PCA group legends: Br-April (Autumn), the month of April is mid-Autumn in Brazil; Br-Oct (Spring), October is early Spring in Brazil; Br-Dec (Autumn), the month of December is late-Spring in Brazil. Loading variables and metabolites: **1**, kaempferol-3-*O*-dihexoside; **2**, caffeoyl glucoside; **3**, 3-*O*-caffeoylquinic acid; **4**, 5-*O*-caffeoylquinic acid; **5**, kingiside; **6**, 8-epi-kingisidic acid; **7**, (7*α*)-7-*O*-methylmorroniside; **8**, (7*β*)-7-*O*-methylmorroniside; **9**, alpigenoside; **14**, fatty (linolenic) acid.

In technical terms, PCA reduces the multidimensional data, such as the transformation of NMR spectra to fewer principal components (PCs) that delineate informative picture of the spectral data. For instance, ^1^H HR-MAS NMR dataset driven by PCA has supported quality control, inter- to intra-plant, and the metabolic variations triggered by environmental factors^3,17,18^. Herein, evaluating metabolic link with seasonal change, the ^1^H HR-MAS NMR dataset was assayed in PCA (Fig. 4).

The given PCA model (Fig. 4) comprised 27 ^1^H HR-MAS NMR profiles from *C. gongonha* Mart. In general consideration of harvest to analyses, sampling was accomplished in Oct and Dec (2018), and April (2019). Somehow, the analysis of PCA scores (Fig. 4) revealed grouping for complete NMR profiles. The NMR data on post-scattering between PC1 (59.5%) and PC2 (18.5%), presented net variance of 78.0% by using Pareto scaling. Throughout, samples in Oct and Dec were pooled in a large group separated from those in April as a small group. Analysis of large cluster confirmed a surplus drift for the samples in Oct, which fairly discrete in negative PC1 and positive PC2. Samples (sub)grouping emphasizes that some irregularity has occurred along the seasons, visibly in spring (Oct to Dec) and autumn (April), whereas spring starts in Oct and ends at Dec in Brazil. Beyond a seasonal aspect, data dispersal can also indicate surplus ecological interactions that trigger metabolites profile in the plant.

**Figure 5:**
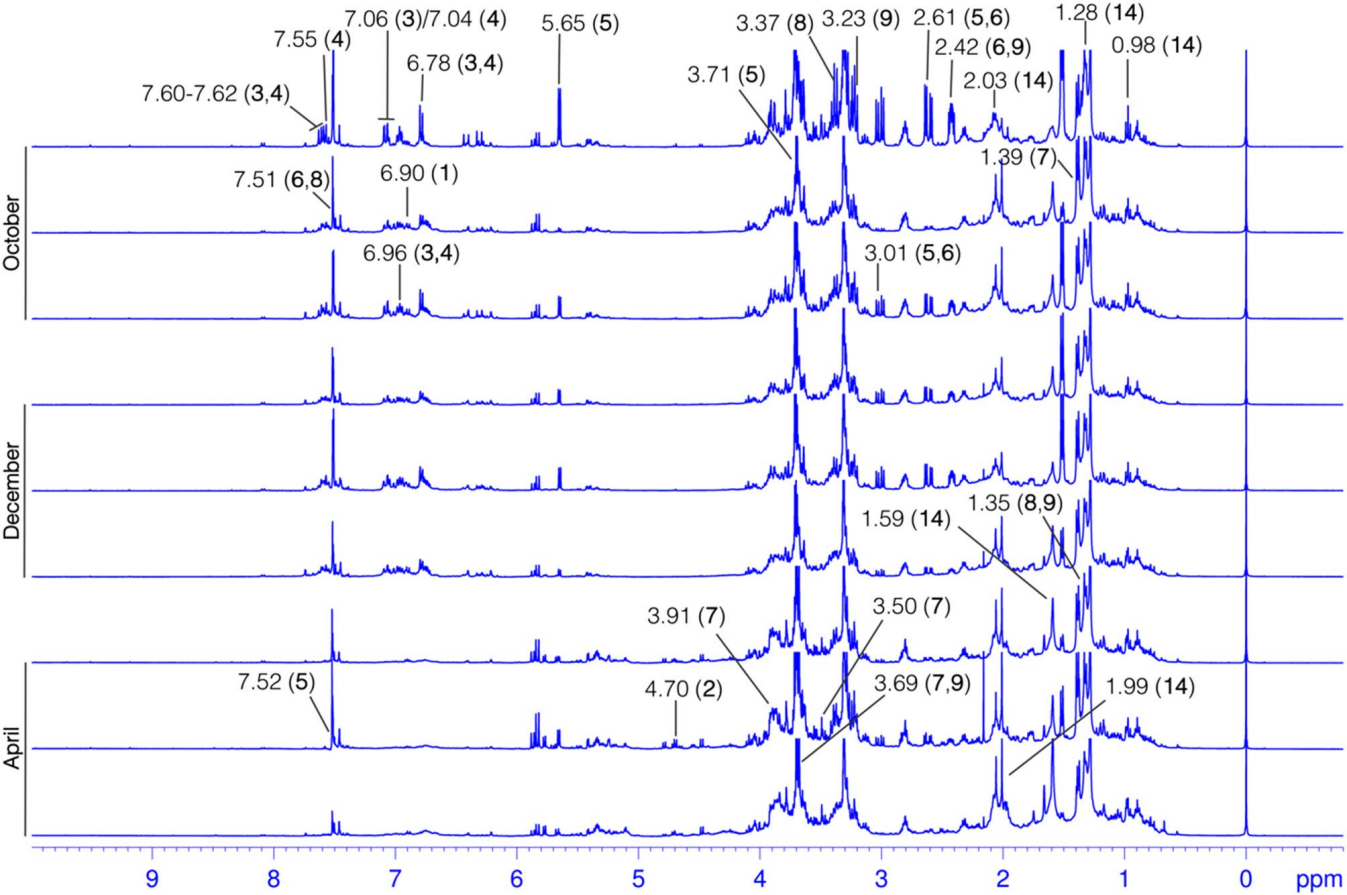
Stacked spectra of ^1^H HR-MAS NMR exhibiting peaks of the discriminatory metabolites in comparison with PCA. NMR data were processed in TopSpin v3.6.3 software package and preprocessed in analysis of mixture (AMIX) v3.9.12 software package (Bruker BioSpin: https://www.bruker.com), PCA analysis was performed in the MetaboAnalyst v5.0 (https://www.metaboanalyst.ca/). Representations for discriminatory metabolites indicating main variables in the loading plot were manually produced in Microsoft PowerPoint v16.56 (https://officecdnmac.microsoft.com), and the GIMP v2.10.24 software package (https://www.gimp.org) was used for final figure generation. PCA group legends: Br-April (Autumn), the month of April is mid-Autumn in Brazil; Br-Oct (Spring), October is early Spring in Brazil; Br-Dec (Autumn), the month of December is late-Spring in Brazil. Loading variables and metabolites: **1**, kaempferol-3-*O*-dihexoside; **2**, caffeoyl glucoside; **3**, 3-*O*-caffeoylquinic acid; **4**, 5-*O*-caffeoylquinic acid; **5**, kingiside; **6**, 8-epi-kingisidic acid; **7**, (7*α*)-7-*O*-methylmorroniside; **8**, (7*β*)-7-*O*-methylmorroniside; **9**, alpigenoside; **14**, fatty (linolenic) acid.

Analyzing metabolites profile, all (sub)groups can be differentiated on the basis of sugar and a set of derivatives of kaempferol-3-*O*-dihexoside (**1**), caffeoyl glucoside (**2**), 3-*O*-caffeoylquinic acid (**3**) and 5-*O*-caffeoylquinic acid (**4**), kingiside (**5**), 8-epi-kingisidic acid (**6**), (7*α* and 7*β*))-7-*O*-methylmorroniside (**7** and **8**), alpigenoside (**9**), and the content of fatty (linolenic) acid (**14**). These distinguishing metabolites in PCA (sub)groups (Fig. 4) are in agreement with the ^1^H HR-MAS NMR result (Fig. 5). The variables in PCA loading are equivalent to ^1^H HR-MAS NMR peaks or chemical shifts of the protons in individual metabolites. To better apprehend seasonal and environmental correlation with (sub)groups and metabolites, a conjoined analysis of PCA (Fig. 4) and ^1^H HR-MAS NMR (Fig. 5) is highlighted.

In accordance with PCA analysis, samples in a large group were described by several NMR peaks, representing the protons in sugar, metabolites **1**–**9**, and **14**. ^1^H HR-MAS NMR (Fig. 5) exposed that sugar (δ_H_ 3.77) along with **1** allowed group discernment on the basis of aromatic ring protons of H-3′/H-5′ (δ_H_ 6.90, m). Group classification by metabolites **2** and **3** was established through a vinylic proton of H-7 [(δ_H_ 7.60, d, *J* = 15.9 Hz), H-7 (δ_H_ 7.62, d, *J* = 15.9 Hz)]. Further peaks from **3** included the aromatic ring protons of H-2 (δ_H_ 7.06, d, *J* = 2.0 Hz), H-5 (δ_H_ 6.78, d, *J* = 8.2) and H-6 (δ_H_ 6.96, dd, *J* = 8.2; 2.0 Hz), while several other from **4** incorporated the aromatic ring protons of H-2 (δ_H_ 7.04, d, *J* = 2.0 Hz), H-5 (δ_H_ 6.78, d, *J* = 8.2), H-6 (δ_H_ 6.96, dd, *J* = 8.2; 2.0 Hz) and vinylic proton of H-7 (δ_H_ 7.55, d, *J* = 15.9 Hz) that allowed group differentiation. Metabolite **5** led a same group identification through the pyran ring proton of H-1 (δ_H_ 5.65, d, *J* = 6.1 Hz), methylene protons of H-6a/H-6b [(δ_H_ 3.01, dd, *J* = 17.1; 6.0 Hz)/(δ_H_ 2.61, dd, *J* = 17.1; 6.0 Hz), methyl protons of H-10 (δ_H_ 1.52, d, *J* = 6.8 Hz/δ_H_ 1.50–1.53) and methoxy protons of H-12 (δ_H_ 3.71, s). Conversely, metabolite **6** permitted group distinction due to the pyran ring proton of H-3 (δ_H_ 7.51, s), methylene protons of H-6a/H-6b [(δ_H_ 3.01, dd, *J* = 17.1; 6.0 Hz)/(δ_H_ 2.61, dd, *J* = 17.1; 6.0 Hz), H-9 (δ_H_ 2.42, dddd *J* = 13.6; 10.2; 6.1; 4.1 Hz) and methoxy protons of H-10 (δ_H_ 1.52, d, *J* = 6.8 Hz/δ_H_ 1.50–1.53). Similarly, **7** was a group discriminatory compound, exhibiting a proton of H-10 (δ_H_ 1.39, d, *J* = 6.9 Hz), along with the pyran ring proton of H-3 (δ_H_ 7.51, s) and methyl protons of H-10 (δ_H_ 1.52, d, *J* = 6.8 Hz/δ_H_ 1.50–1.53) from metabolite **8**. In turn, the discriminatory protons from metabolite **9** were H-5 (δ_H_ 3.23, m) and H-6a (δ_H_ 2.42, dddd *J* = 13.6; 10.2; 6.1; 4.1 Hz). Also, **14** distinguished group because of H-8/H-17 (δ_H_ 2.03, m) and H-18 (δ_H_ 0.98, t, *J* = 7.5 Hz).

Consistently, samples in the April group were distinguished by metabolite **2** due to proton in the *β*-D-glucose unit such as *β*-H-1′ (δ_H_ 4.70, d, *J* = 7.9 Hz). Equally, compound **5** permitted group distinction by pyran ring proton of H-3 (δ_H_ 7.52, s), while H-7 (δ_H_ 3.50, s) and H-8 (δ_H_ 3.91, m) were from compound **7**. In addition to H-10 (δ_H_ 1.35, d, *J* = 6.9 Hz) from **8** and **9**, the proton of H-11 (δ_H_ 3.69, s) was typically from metabolites **7** and **9**. Samples in the same group were further illustrated by sugar (δ_H_ 3.53 to 3.56).

In context, samples in Oct showing negative value of PC1, were illustrated by additional chemical shifts associated to protons of H-8/H-17 (δ_H_ 2.03, m), H-18 (δ_H_ 0.98, t, *J* = 7.5 Hz) and H-4 to H-7 (δ_H_ 1.28, br d) from fatty (linolenic) acid (**14**), and similarly H-10 (δ_H_ 1.39, d, *J* = 8.9 Hz) from compound **7**. However, samples in the positive PC2 were distinguished through the peaks from protons in metabolites **5**, **6** and **8**—e.g., H-6a (δ_H_ 3.01, dd *J* = 17.1; 7.6 Hz) of **5** and **6**, H-1 (δ_H_ 5.65, d, *J* = 6.1 Hz) and H-12 (δ_H_ 3.71, s) of **5**, while H-7 (δ_H_ 3.37, s) of metabolite **8**. According to ^1^H HR-MAS NMR (Fig. 5), group discriminatory peaks specified an increased relative intensity, if compared in all spectra from relevant intervals. To this end, these irregularities in NMR peaks express not only seasonal or environmental effects^3,14,17,31^ but can also display the elaboration of other interactions^32^.

In general, according to spring, samples deviation was evidently the effect on sugar, kaempferol and phenolic compounds (**1**–**4**), monoterpenoids or secoiridoids (**5**–**9**), and fatty acid (**14**). But in particular, the early-spring (Oct), which is a dry period, caused samples variability mainly due to metabolites **5**, **7** and **14**. In relevant period, an intra-plant variability is greater because of the influence of irregularity in climatic conditions that can include sunlight, temperature, precipitation rate and rainfall, etc. In contrast, in late-spring (Dec), due to constancy in temperature and reduced rainfall, leaf metabolic profiles are equivalent. Effect on the samples in autumn was correlated with metabolites **2**, **5**, **7**–**9** and **14**, however this is also dry period with low environmental temperature and rainfall. Besides that, there can be many more interactions that triggered leaf metabolic patterns. Following seasonal impact^33^, the evolution of the given metabolites illustrates several climatic stresses for example drought, sunlight, rainfall, humidity, nutrients availability, amongst others^32,34,35^. A dry period—e.g., Oct and April, has displayed significant relation with the sugar, suggesting that this period stimulates the production of relevant content in plants. Relatedly, fatty (linolenic) acid (**14**) showed correlation with samples in almost both seasons. This chemical compound is useful in varied mechanisms, involving the protection of plant in severe state; therefore, the accumulation of **14** denotes either seasonal or more possible interactions^36^. However, the accumulation of compounds **1**–**4** or secoiridoids **5**–**9** can be correlated to effects surging from the stated factors. Besides seasonal adaptation, temperature and rainfall have been major inducers, indicating a positive correlation with plant phenolic compounds^34^. In this scenario, drought has been a major impacting factor on plant phenolic content together with the overall growth of plant^37^. The accumulation of compounds **5**–**9** can be related with the effect of sunlight and nutrients availability, etc. In harmony, the derivates of plant iridoids and phenolic metabolites have been studied, and found that the production of such compounds have significant correlation with the light and nutrients availability^38^. The accumulation of these metabolites can also suggest the result of the soil that contains, for example an increased level of aluminum^39^. A great part of soil composition in Paraná state, Brazil can contain a high level of aluminum. Irregularity in temperature is a fundamental trend, which has a renowned influence on plant physiology, development, metabolism and metabolites profile^40^. Nevertheless, temperature has synchronizing role in many chemical reactions that can incline or decline progress of plant metabolites. On the other hand, light can be another factor that has known impact on plant growth and the quantitative profile of chlorogenic acid or cinnamic acid^41^.

## Conclusion

This study determined the metabolites profile of leaf part from *C. gongonha* Mart. (Cardiopteridaceae). The diversity of metabolites detected by ^1^H HR-MAS NMR and LC–MS/MS provides to this plant a high medicinal potential, as indicated by the abundant quantities of the phenolics and monoterpenoid compounds found in the leaf extracts. In line with this knowledge, a mutual correlation of metabolites with seasonality or climatic conditions was assessed in PCA, exposing that many metabolites were influenced in early-spring and autumn. These results demonstrate that *C. gongonha* Mart. is more susceptible to seasonal adaptation and environmental factors (e.g., drought, sunlight, temperature, etc.). Because this work analyzed a small sample size in two seasons, further studies should involve large sample size with respect to all seasons. The ecological parameters (https://www.sanepar.com.br/) greatly vary day-by-day in individual seasons of the year in Curitiba-Paraná, Brazil. Therefore, it can be suggested to replicate these findings for more evaluation of the same plant at diverse geographical sites. Through these findings, we further recommend evaluating this plant under controlled environmental conditions, to understand the physiology and mechanisms involved in the production of these and new metabolites.

## Material and methods

### Experimental methods

In the present work, liquid nitrogen (N_2_) was used to grind the leaf samples manually with a mortar and pestle. The entire measurements presented herein were performed in deuterated methanol (CD_3_OD, 99.8% D; TMS, 0.05%) purchased from the Cambridge Isotope Laboratories, Inc., Andover, MA, USA. The semisolid-state ^1^H HR-MAS and 2D NMR analyses in solution-state were acquired on a Bruker AVANCE 400 NMR spectrometer (Bruker BioSpin, Karlsruhe, Germany) operating at 9.4 Tesla (^1^H = 400.13 MHz; ^13^C = 100.6 MHz). ^1^H HR-MAS NMR acquisition with a standard *zgcppr* pulse sequence (Bruker library) was performed by a four-channel (^1^H, ^13^C, ^15^N, and ^2^H) 4-mm HR-MAS probe with gradient field in a magic angle (θ = 54.74°) direction to the externally applied, static magnetic field (B_0_). Whereas, in 2D NMR, a 5-mm broadband inverse detection four-channel (^1^H, ^2^H, ^13^C, and ^31^P) probe was used with magnetic field gradient along the z-axis. Solution-state acquisitions were carried out with standard pulse sequences from the Bruker library that included multiplicity-edited HSQC and HMBC to assess ^1^H-^13^C single-bond (^1^J_H-C_ = 145 Hz) to long-range (^LR^J_H-C_ = 8 Hz) correlations, while ^1^H-^1^H correlations were measured with the 2D double quantum filter (DQF-)COSY NMR. The LC–MS/MS method was implemented to confirm chemical structures of some chemical compounds. Such analyses were completed for the sample ethanolic extract (70% ethanol*,* HPLC grade and 30% water, Milli-Q).

### Leaf sampling

The collection of the plant specimen in this study was approved by the department of chemistry and the department of botany, polytechnic center at Federal University of Paraná (Municipal Decree no. 170/15) by conforming the national and international guideline and legislation. In this way, healthy and mature leaves were harvested from adult *C. gongonha* Mart., in an open field. Samples collection was completed in a period of two seasons (early October to December 2018 (spring)—early April 2019 (autumn)) at Curitiba, PR (5°27′11″S × 49°14′06″W) Brazil. Throughout, post-sampling and cleansing with water, the leaves were dried in circulating air (45 °C) in the oven for 48 h, and then stored under a freezing temperature (− 18 °C) until the NMR analyses. Plant specimen was taxonomically identified and deposited under voucher no. MBM 415085^3^, in the botanical (Museu Botânico Municipal: MBM) Herbarium of Curitiba, PR, Brazil.

### Sample preparation

In ^1^H HR-MAS NMR acquisitions, 10.00 ± 0.05 mg ground leaf was filled in a 50 µL zirconium (ZrO_2_) magic angle spinning (MAS) rotor (Bruker, BioSpin) and 40 μL CD_3_OD were added. To attain better stability for B_0_ through shimming, the materials in the MAS rotor were homogenized, and air bubbles were removed with a syringe needle. After tight packing of the sample in the MAS rotor, the sample was left in contact with CD_3_OD for 18 min, and then submitted for acquisitions. The extract, for 2D NMR spectroscopy, was prepared by taking 100.0 ± 1.0 mg powdered leaf in a microcentrifuge tube (1000 µL), with a 600 µL CD_3_OD. Post-sonication for 40 min (25 °C), the mixture was centrifuged at 12,000 rpm for 30 min (Microcentrifuge, MCD2000). When centrifuged, a 500 µL methanolic phase was transferred into a 5-mm NMR tube for analysis. Powdered leaf sample of 1000 mg was prepared in a 10,000 μL of 70% ethanol (HPLC grade) and left overnight on magnetic agitation. The extract after filtration in a 0.45 μL syringe, was submitted to the LC–MS/MS analysis.

### ^1^H HR-MAS and 2D NMR measurements

In semisolid-state ^1^H HR-MAS NMR, the spinning frequency of the MAS rotor was adjusted to 5000 Hz at a 296 K temperature. The magic angle (θ = 54.74°) tuning was completed manually, by a reference signal of ^79^Br from standard KBr. Prior and post-manual homogeneity of B_0_, tuning, and matching was constantly directed to a proton (^1^H) frequency. ^1^H HR-MAS NMR acquisitions were conducted with standard *zgcppr* pulse sequence (Bruker, library) to saturate strong resonance from water in the samples. The acquisition parameters for *zgcppr* encompassed a free induction decay (FID) size of 64,000 data points, sweep width (SW) of 8012.82 Hz, acquisition time (AQ) of 4.1 s, FID resolution of 0.24 Hz, transmitter offset frequency (O1) of 1949.76 Hz, the temperature of 296 K, recycle delay (D1) time of 1 s, pre-saturation power (pl9) of 55 dB, and radiofrequency pulse (B_1_) duration of 5.63 µsec with total 256 scans (NS). Spectral processing was performed by exponential window multiplication to the FIDs, using a Lorentzian line broadening function (LB) to 0.3, and zero-filled to 64,000 data points. ^1^H HR-MAS NMR spectra were referenced to the tetramethylsilane (TMS signal at δ_H_ 0.00). Except for a magic angle adjustment, the total experimental time was 22 min, including preparation, rotor cleaning and packing, tuning, matching, and shimming for each measurement, respectively.

### LC–MS/MS measurements

Sample analyses were performed with a Waters Acquity I-Class UPLC system (Waters, Milford, MA, USA) equipped with a BEH C18 column (2.1 × 100 mm, 1.7 μm) coupled to a Xevo TQ-S triple quadrupole mass spectrometer (Waters, Milford, MA, USA). For chromatographic separation, a 5 μL diluted leaf ethanolic extract was injected into the LC–MS/MS system. During analyses, the column was maintained to 40 °C, while the sample was kept at 15 °C. Mobile phase of water (eluent A) and acetonitrile (eluent B) were contained 0.1% formic acid. For elution of injected sample, the percentage of eluent A was 95% for 0.5 min. Followed that, eluent B was linearly raised to 7% over 1.00 min, yet maintained 10% for 1.0–2.0 min. Eluent B was increased to 60% over 11.5 min, followed by a hold time of 0.5 min. Then, eluent B was increased to 95% over 0.5 min, and column was stabilized to reach initial conditions (5% B) for 0.5 min. Chromatographic run time was 15 min, and the mobile phase flow rate was maintained to 0.4 mL min^−1^. The equipment was operated in single ion reaction (SIR) and MS^2^ modes using electrospray ionization (ESI) in positive and negative ion modes. All experimental parameters included: capillary voltage: 3.50 kV; source temperature: 150 °C; desolvation temperature: 400 °C, desolvation gas flow: 800 Lh^−1^; cone gas flow: 150 Lh^−1^. Cone voltage and collision energy were manually optimized for individual metabolites. Data collection and processing were performed in MassLynx v.4.1 software.

### Principal component analysis (PCA)

The entire ^1^H HR-MAS NMR dataset for *C. gongonha* was recorded in triplicate (*n* = 3) from different parts within the same plant, according to our previous studies^3,17^. Spectral baselines and phases were manually corrected, and the chemical shifts were referenced (TMS signal at δ_H_ 0.00) in TopSpin v. 3.6.3 software package (Bruker BioSpin). The data was preprocessed in the analysis of mixture (AMIX) v. 3.9.12 software package (Bruker BioSpin). Considering the frequency range of δ_H_ 0.56–8.15, several regions without signals (< δ 0.55 and > δ_H_ 8.16), residual water (δ_H_ 4.90–5.05), and methanol (δ_H_ 3.29–3.32) were excluded. Then the NMR dataset was binned into a table, using a bin size of δ_H_ 0.03. At this stage, the generated bucket table of 27 (samples in rows) × 264 (chemical shifts as columns) was transferred into a readable “.txt format”, and submitted to MetaboAnalyst v.5.0, a web-based software (https://www.metaboanalyst.ca/) platform for PCA analysis^42,43^. It was applied a Pareto scaling into the bucket table before the final analysis. All responsible metabolites for score groups were discriminated by means of precise variables in the loadings plot in the PCA model.

## Supplementary Information

Supplementary Information.

## Data Availability

All data generated or analyzed during this study are included in this published article (and its supplementary information file).
