# Supplementary material for: Leaf tissue metabolomics fingerprinting of Citronella gongonha Mart. by 1H HR-MAS NMR
Source: Sci Rep. 2022 Oct 21;12:17624. doi: 10.1038/s41598-022-22708-w (PMC9587042; doi:10.1038/s41598-022-22708-w)
Supplement: Supplementary file 1 — Supplementary Information. [file 41598_2022_22708_MOESM1_ESM.docx]

**Leaf tissue metabolomics fingerprinting of *Citronella gongonha* Mart. by ^1^H HR-MAS NMR**

**Sher Ali^1,2*^, Gul Badshah^3^, Umar Ali^4^, Muhammad Siddique Afridi^5^, Anwar Shamim^6^, Ajmir Khan^7^, Frederico Luiz Felipe Soares^8^, Leociley Rocha Alencar Menezes^1^, Vanessa Theodoro Rezende^9^, Andersson Barison^1^, Carlos Augusto Fernandes de Oliveira^2^ and Fernando Gustavo Tonin^10^**

^1^ Federal University of Paraná (UFPR), NMR Center, Department of Chemistry, Curitiba, PR 81530-000, Brazil

^2^ University of São Paulo (USP), Department of Food Engineering, Faculty of Animal Science and Food Engineering (FZEA), Pirassununga, SP 13635-900, Brazil

^3^ Federal University of Paraná (UFPR), Laboratory of Polymers and Catalysis (LaPoCa), Department of Chemistry, Curitiba, PR, 81530-000, Brazil

^4^ University of Malakand (UoM), Department of Physics, Dir (L), KPK 18800, Pakistan

^5^ Federal University of Lavras, (UFLA), Department of Plant Pathology, Lavras, 3037,37200-900, Brazil

^6^ University of São Paulo – São Carlos Institute of Chemistry (IQSC-USP), Group of Medicinal & Biological Chemistry, São Carlos, SP 13566-590, Brazil

^7^ Michigan State University (MSU), School of Packaging, 448 Wilson Road, East Lansing MI 48824-1223, USA

^8^ Federal University of Paraná (UFPR), Data Science in Chemistry Laboratory, Department of Chemistry, Curitiba, PR 81530-000, Brazil

^9^ University of São Paulo (USP), Department of Animal Science, Faculty of Veterinary and Animal Science (FMVZ), Pirassununga, SP 13635-900, Brazil

^10^ University of São Paulo (USP), Department of Biosystems Engineering, Faculty of Animal Science and Food Engineering (FZEA), Pirassununga, SP 13635-900, Brazil

* **Correspondence:** [alisher@usp.br](mailto:alisher@usp.br) (S.A)

**
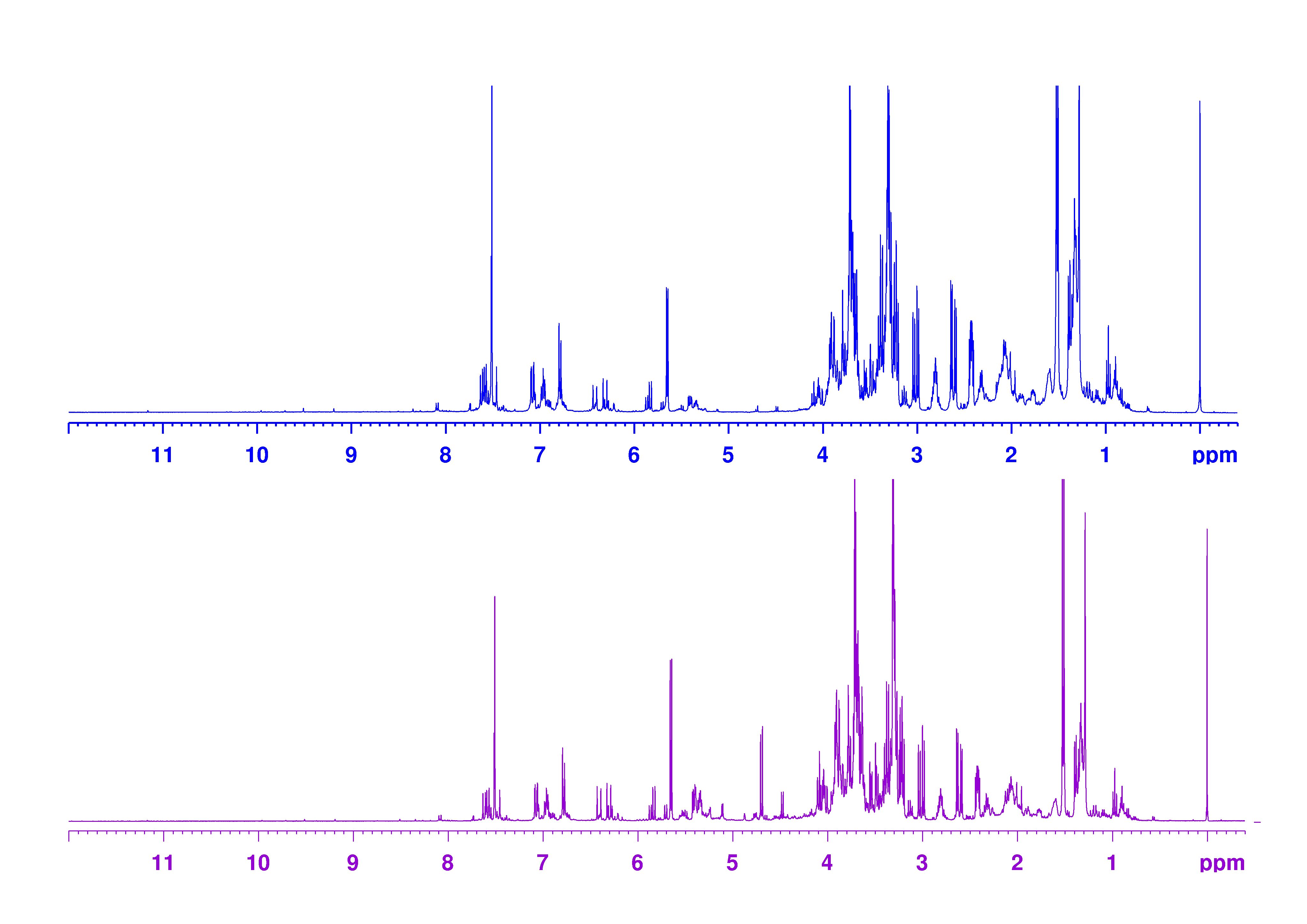
**

**Figure S1.** Comparative overview of 400.13 MHz ^1^H HR-MAS NMR (upper) of leaf tissue (10 ± 0.05 mg swollen in 40 µL CD_3_OD) and ^1^H NMR (lower) of leaf extract (100 ± 1.0 mg in 500 µL CD_3_OD) from *Citronella gonogonha* Mart. Figure was produced in TopSpin v.3.6.3 software package (Bruker BioSpin: <https://www.bruker.com>).

**
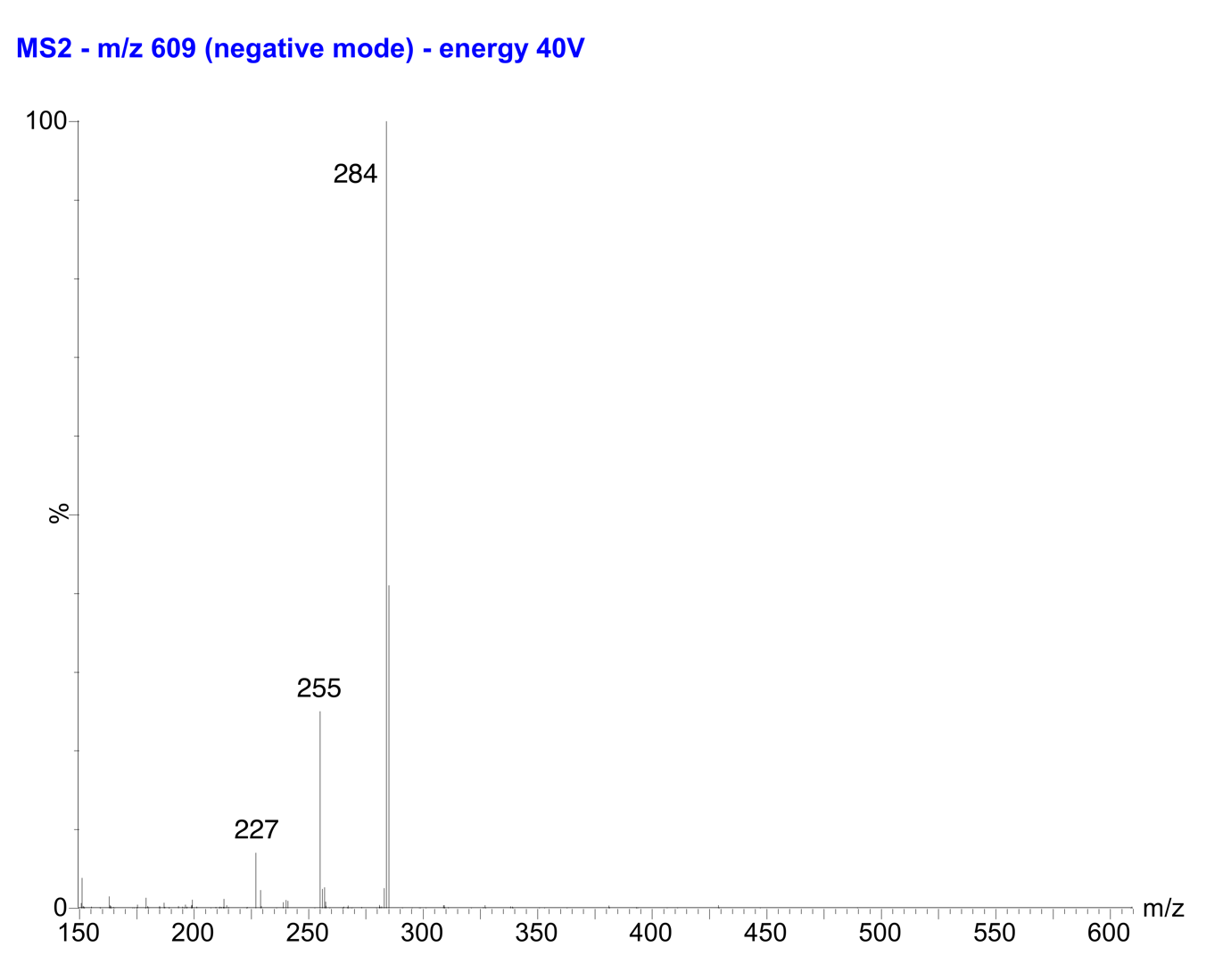
**

**Figure S2.** Mass spectrum of kaempferol-3-*O*-dihexoside (**1a**) in the negative ion mode.

**
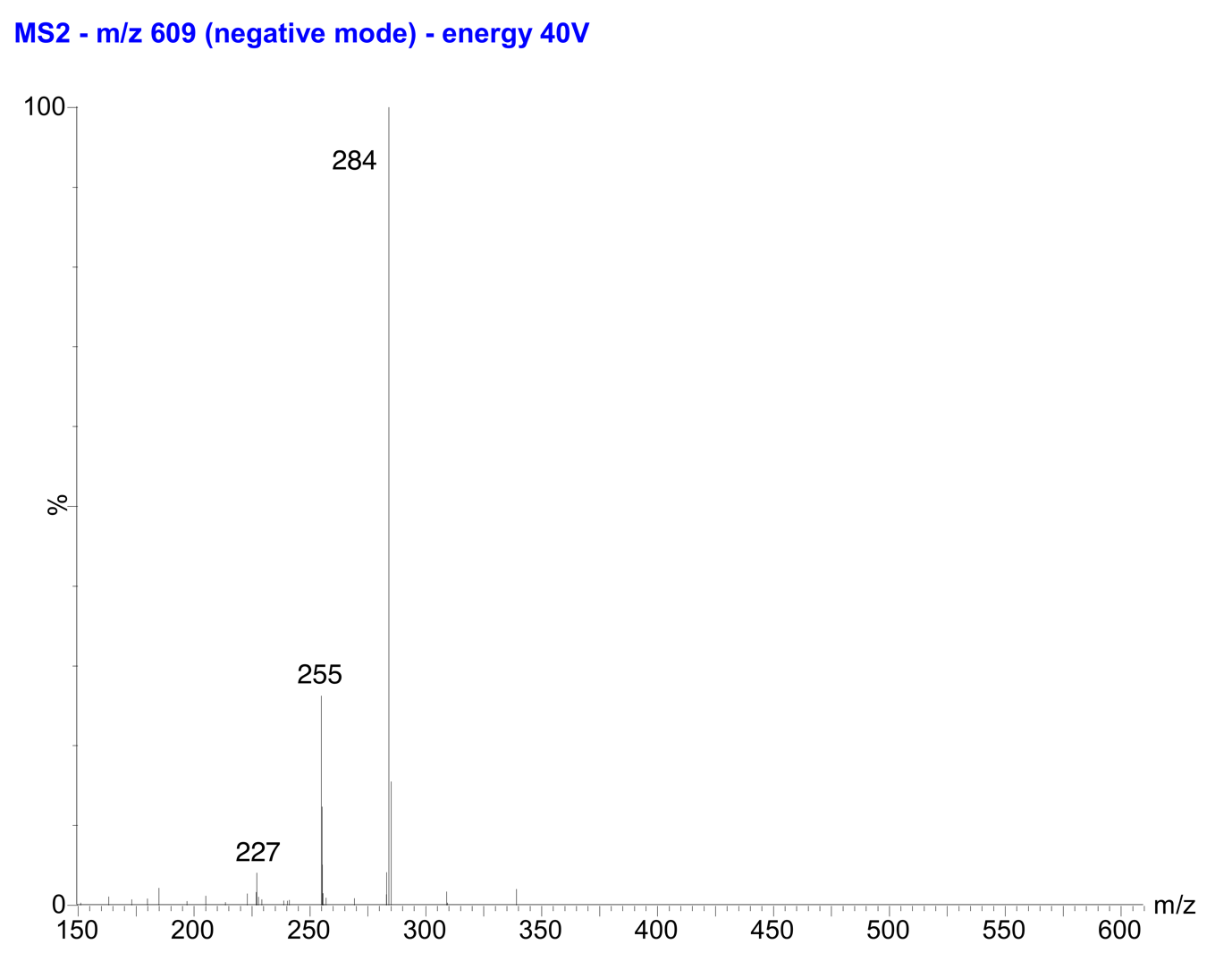
**

**Figure S3.** Mass spectrum of kaempferol-3-*O*-dihexoside (**1b**) in the negative ion mode.

**
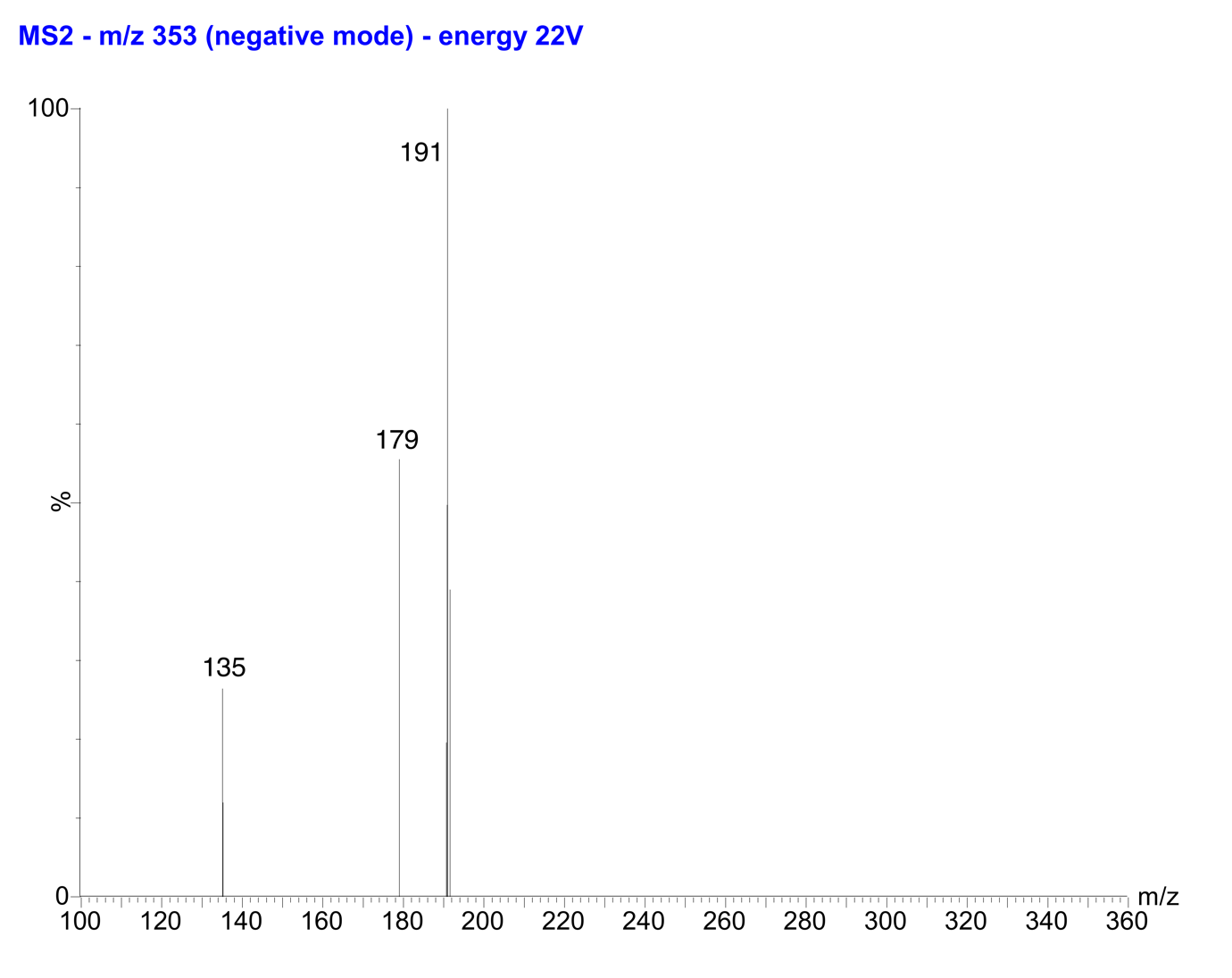
**

**Figure S4.** Mass spectrum of 3-caffeoylquinic acid (**3**) in the negative ion mode.

**
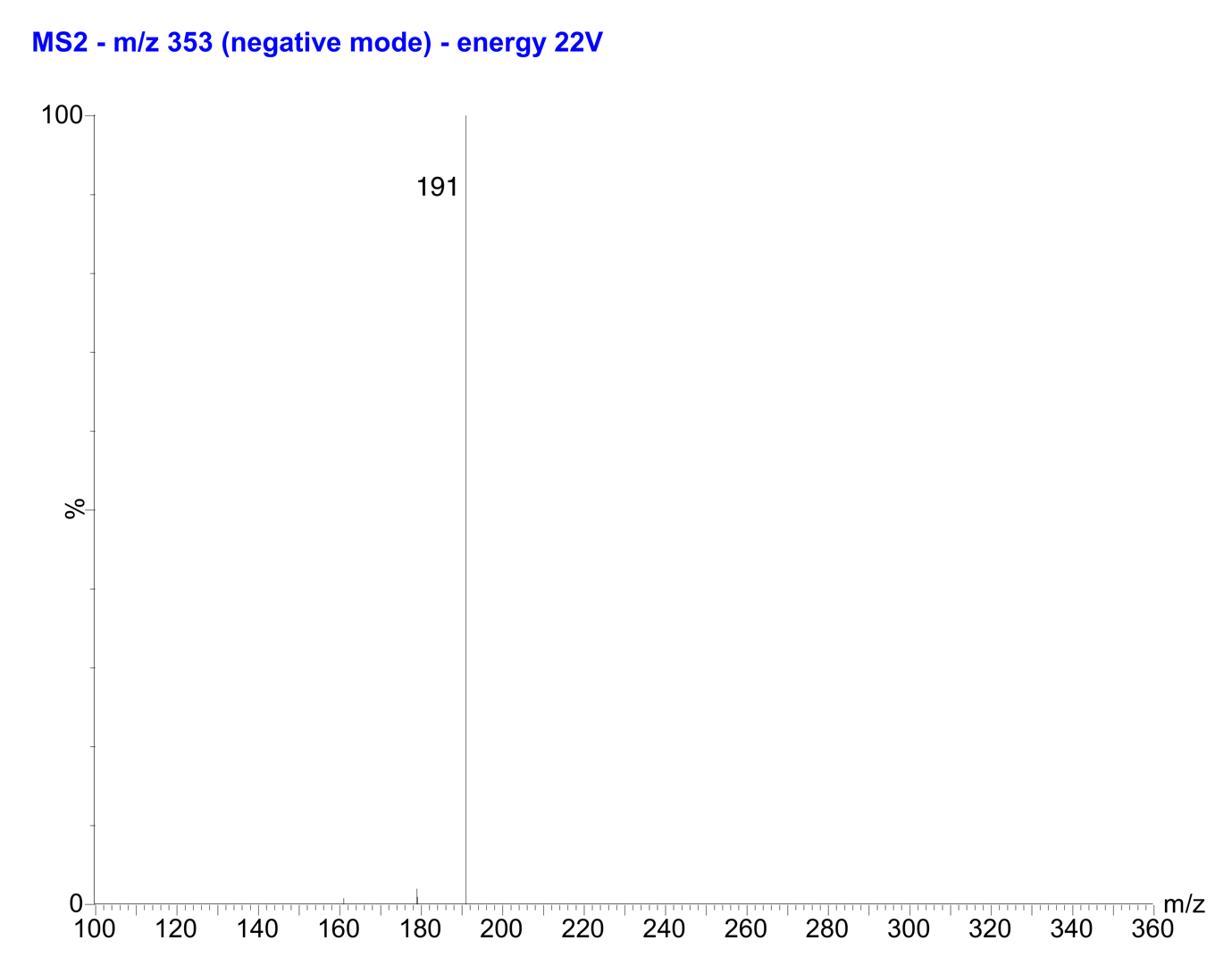
**

**Figure S5.** Mass spectrum of 5-caffeoylquinic acid (**4**) in the negative ion mode.

**
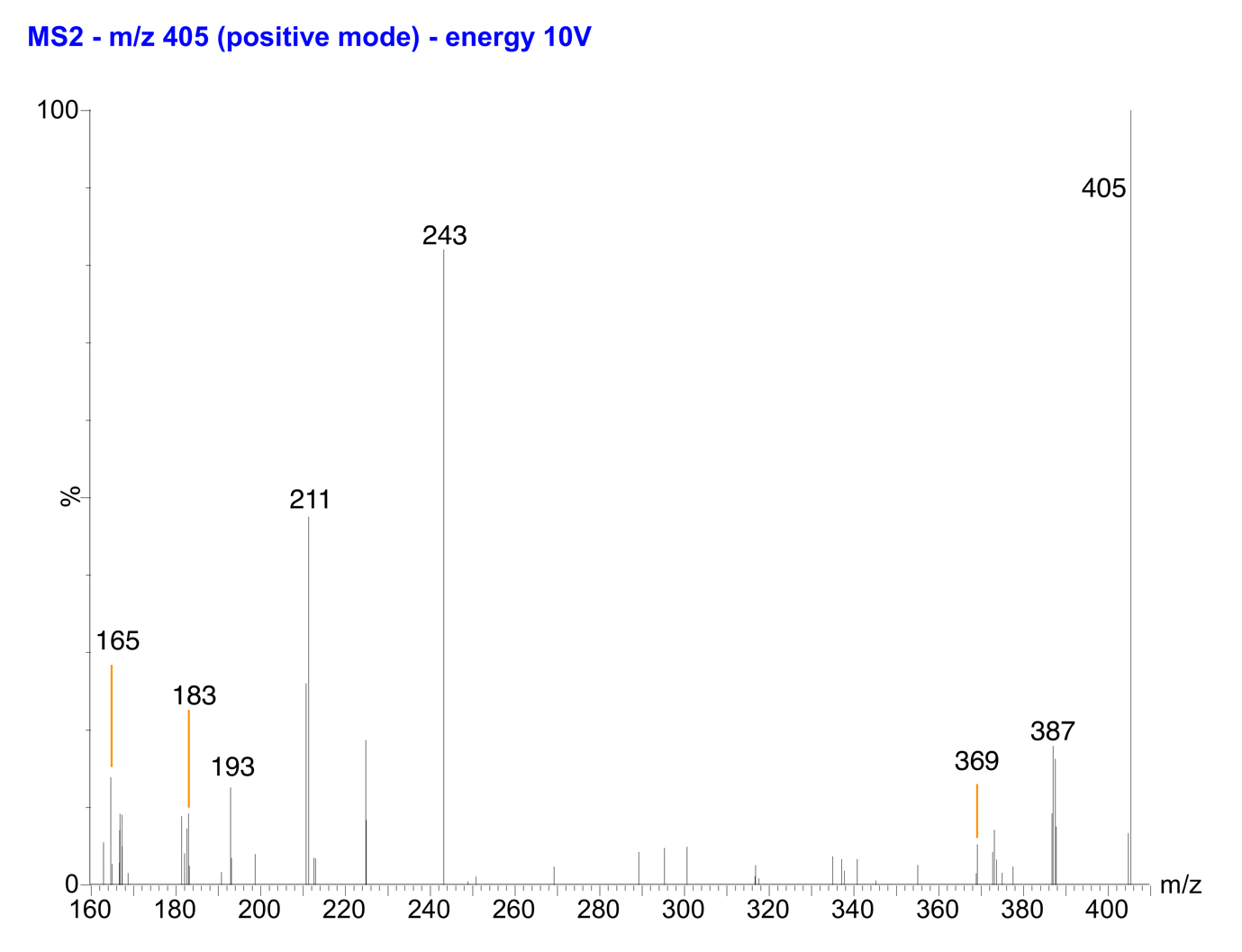
**

**Figure S6.** Mass spectrum of kingiside (**5a**) in the positive ion mode.

**
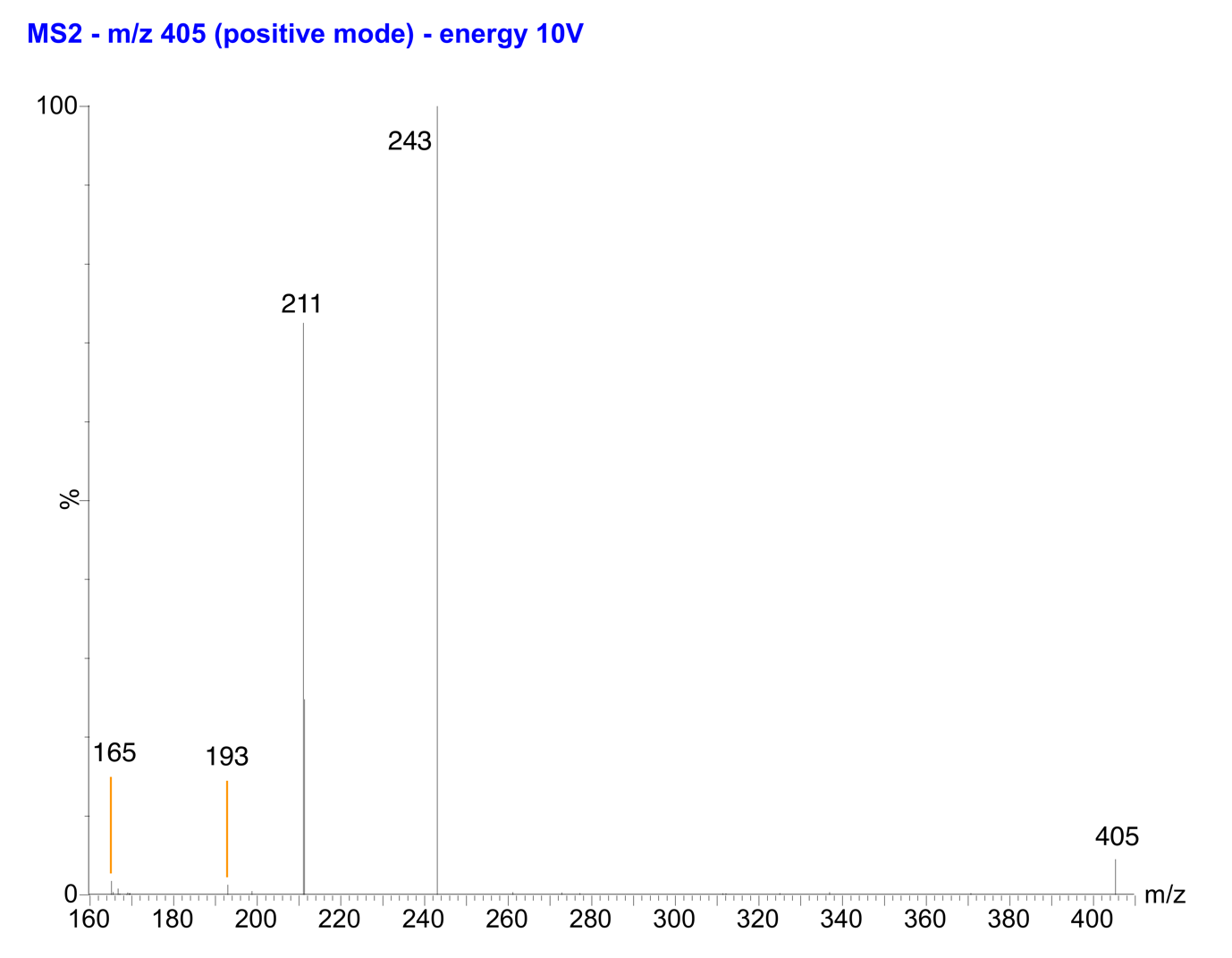
**

**Figure S7.** Mass spectrum of kingiside (**5b**) in the positive ion mode.

**
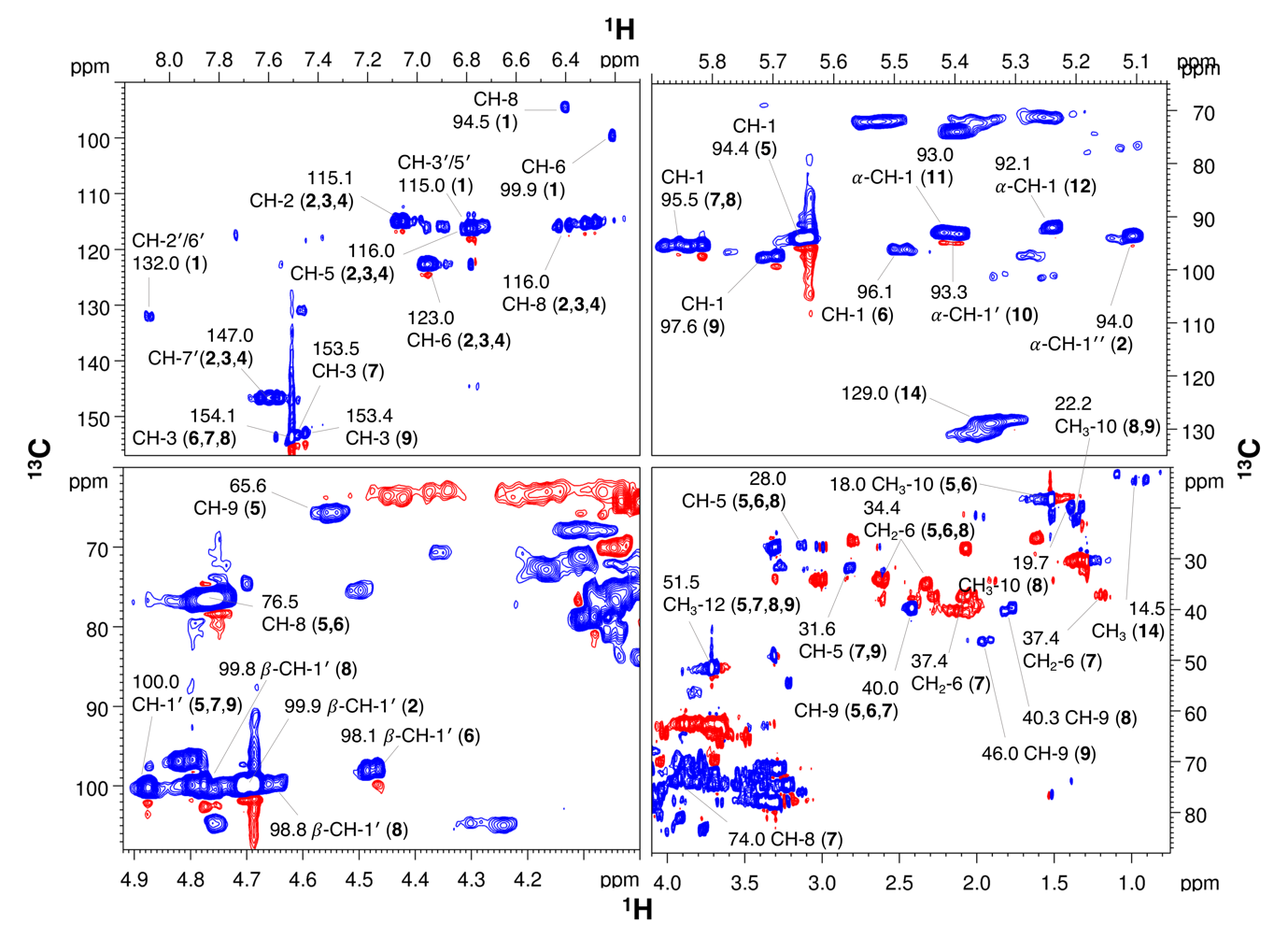
**

**Figure S8.** ^1^H-^13^C direct correlation through multiplicity edited HSQC NMR (^1^H = 400.13/^13^C = 100.6 MHz) of extract from grinded leaf (100 ± 1.0 mg in 500 $\mu$L CD_3_OD) of *Citronella gonogonha* Mart. Blue color signifies CH/CH_3_ and red assignments denote CH_2_. Metabolites: **1**, kaempferol-3-*O*-dihexoside; **2**, caffeoyl glucoside; **3**, 3-*O*-caffeoylquinic acid; **4**, 5-*O*-caffeoylquinic acid; **5**, kingiside; **6**, 8-epi-kingisidic acid; **7**, (7*α*)-7-*O*-methylmorroniside; **8**, (7*β*)-7-*O*-methylmorroniside; **9**, alpigenoside; **10**, sucrose; **11** - **12,** *α*-glucoses; **13**, alanine; **14**, fatty (linolenic) acid. The 2D NMR spectrum was produced in TopSpin v3.6.3 software package (Bruker BioSpin: <https://www.bruker.com>), signal annotations were generated in Microsoft PowerPoint v16.56 (<https://officecdnmac.microsoft.com>), and the final image was prepared in GIMP v2.10.24 software package (<https://www.gimp.org>).

**
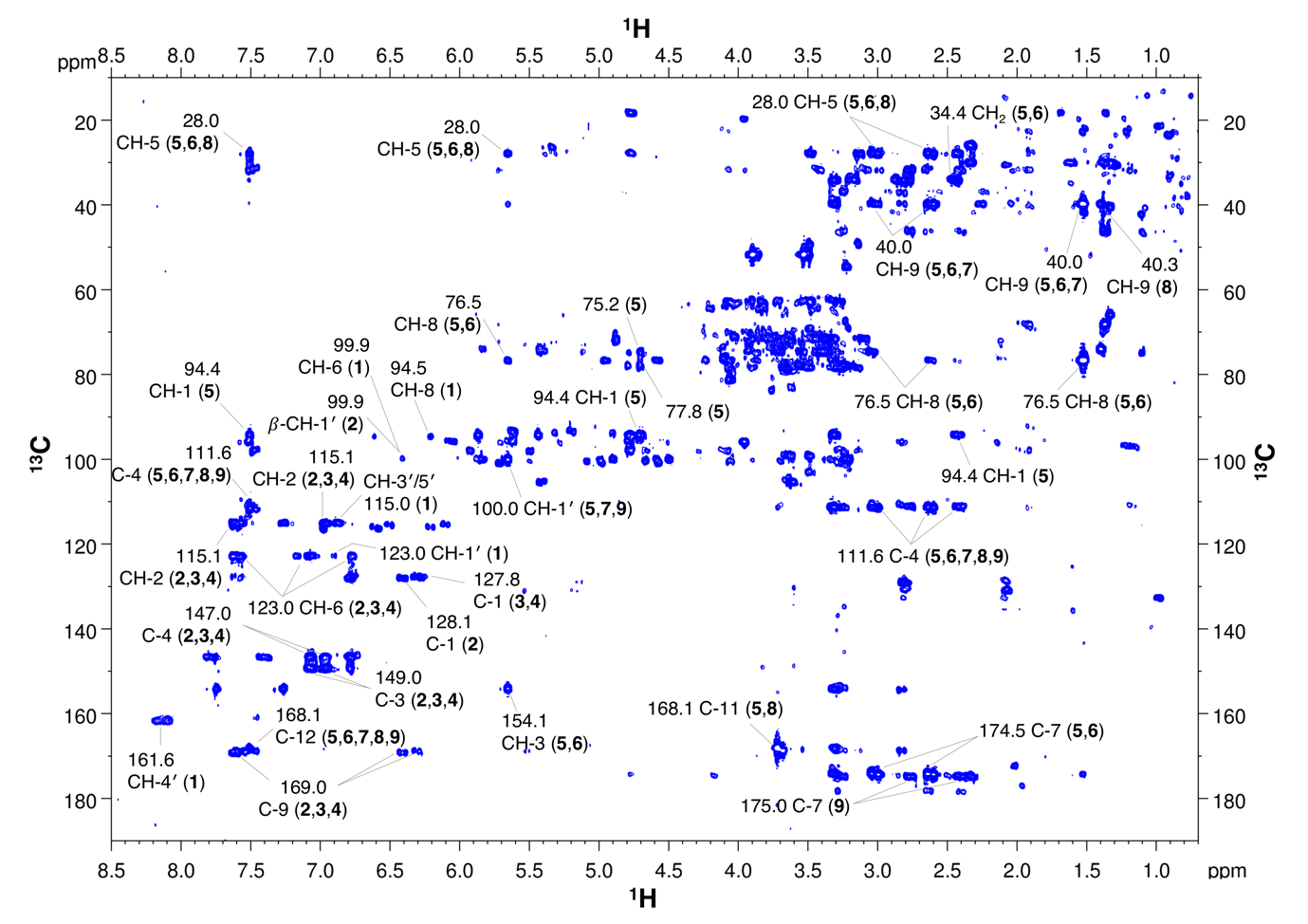
**

**Figure S9.** ^1^H-^13^C long-range correlation from HMBC NMR (^1^H = 400.13/^13^C = 100.6 MHz) of extract from grinded leaf (100 ± 1.0 mg in 500 $\mu$L CD_3_OD) of *Citronella gonogonha* Mart. Metabolites: **1**, kaempferol-3-*O*-dihexoside; **2**, caffeoyl glucoside; **3**, 3-*O*-caffeoylquinic acid; **4**, 5-*O*-caffeoylquinic acid; **5**, kingiside; **6**, 8-epi-kingisidic acid; **7**, (7*α*)-7-*O*-methylmorroniside; **8**, (7*β*)-7-*O*-methylmorroniside; **9**, alpigenoside; **10**, sucrose; **11** - **12,** *α*-glucoses; **13**, alanine; **14**, fatty (linolenic) acid. 2D NMR spectrum was produced in TopSpin v3.6.3 software package (Bruker BioSpin: <https://www.bruker.com>), signal annotations in Microsoft PowerPoint v16.56 (<https://officecdnmac.microsoft.com>), and for final figure the GIMP v.2.10.24 software package (<https://www.gimp.org>) were used.


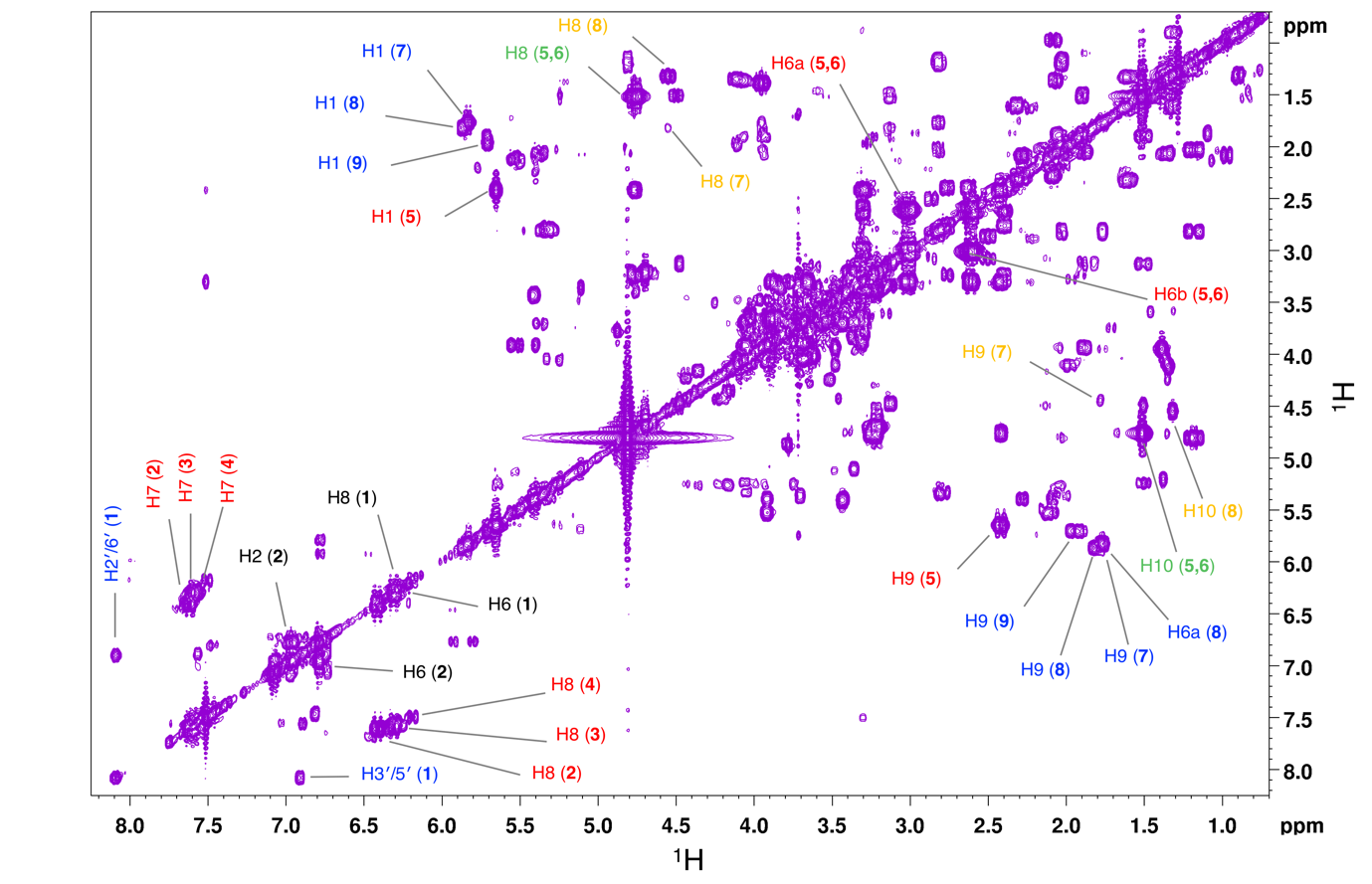


**Figure S10.** ^1^H-^1^H DQF-COSY NMR (^1^H = 400.13 MHz) of powdered leaf extract (100 ± 1.0 mg in 500 µL CD_3_OD) from *Citronella gonogonha* Mart. Metabolites: **1**, kaempferol-3-*O*-dihexoside; **2**, caffeoyl glucoside; **3**, 3-*O*-caffeoylquinic acid; **4**, 5-*O*-caffeoylquinic acid; **5**, kingiside; **6**, 8-epi-kingisidic acid; **7**, (7*α*)-7-*O*-methylmorroniside; **8**, (7*β*)-7-*O*-methylmorroniside; **9**, alpigenoside. The 2D NMR spectrum was produced in TopSpin v3.6.3 software package (Bruker BioSpin: <https://www.bruker.com>), signal annotations were generated in Microsoft PowerPoint v16.56 (<https://officecdnmac.microsoft.com>), and the final image was prepared in GIMP v2.10.24 software package (<https://www.gimp.org>).

**Chemical structures elucidation.** The following chemical structures of major metabolites detected in ^1^H HR-MAS NMR have been discriminated by high-resolution 2D (^1^H-^13^C HSQC and HMBC, and ^1^H-^1^H DQF-COSY) NMR with the support of literature, as described.

**Structural detail for kaempferol-3-*O*-dihexoside (1a, b)**


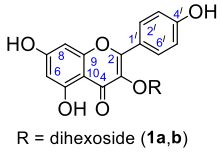


| **Experimental data in comparison with Ref.**^1^ | | | | |
| --- | --- | --- | --- | --- |
| **Metabolite** | **Position** | **δ_H_ (J in Hz)** | **δ_C_ (HSQC)** | (**^LR^J_H-C_**) **HMBC** |
|  | 1 – 5 | - | - | - |
| kaempferol-3-*O*-dihexoside [**1** (**a**, **b**)] | 6 | 6.21 (d = 2.1) | 99.9 | 94.5 |
|  | 7 | - | - | - |
|  | 8 | 6.39 (d = 2.1) | 94.5 | 99.9 |
|  | 1′ | - | 123.0 | - |
|  | 2′/6′ | 8.08 (d = 9.0) | 132.0 | 161.6 |
|  | 4′ | - | 161.6 | - |
|  | 3′/5′ | 6.90 (m) | 115.0 | 123.0; 115.0 |

**Table S1.** ^1^H HR-MAS and 2D NMR-based structure confirmation of kaempferol (**1**) detected in the leaves of *Citronella gongonha* Mart. (Cardiopteridaceae). Experimental data: ^1^H 400.13 and ^13^C 100.6 MHz; CD_3_OD included TMS (*v*/*v*, 0.05%) as reference standard. All chemical shifts (δ) are in ppm and coupling constants (J) are given in Hertz (Hz). Doublet (d), and multiplet (m). Heteronuclear (^1^H-^13^C) single quantum correlation (HSQC). Long-range (^1^H-^13^C) Heteronuclear multiple bond correlation (^L-R^J_H-C_ HMBC).

**Structural detail for caffeoyl glucoside (2)**

| **Experimental data in comparison with Ref.**^2^ | | | | |
| --- | --- | --- | --- | --- |
| **Metabolite** | **Position** | **δ_H_ (J in Hz)** | **δ_C_ (HSQC)** | (**^LR^J_H-C_**) **HMBC** |
| Caffeoyl glucoside (**2**) | 1 | - | 128.1 | - |
|  | 2 | 7.08 (d = 2.0) | 115.1 | 149.0; 147.0; 123.0 |
|  | 3 | - | 149.0 | - |
|  | 4 | - | 147.0 | - |
|  | 5 | 6.77 (d = 8.2) | 116.4 | 128.1; 149.0; 147.0; 123.0 |
|  | 6 | 6.95 (dd = 8.2; 2.0) | 123.0 | 115.1; 149.0; 147.0 |
|  | 7 | 7.61 (d = 15.9) | 146.8 | 115.1; 123.0; 169.0 |
|  | 8 | 6.41 (d = 15.9) | 115.5 | 128.1; 169.0; 99.9 |
|  | 9 | - | 169.0 | - |
|  | 1′ | 4.70 (d = 7.9) | 99.9 | 94.0; 77.7; 75.1 |
|  | 1″ | 5.11 (d = 3.7) | 94.0 | 99.9 |

**Table S2.** ^1^H HR-MAS and 2D NMR-based structure confirmation of caffeoyl glucoside (**2**) detected in the leaves of *Citronella gongonha* Mart. (Cardiopteridaceae). Experimental data: ^1^H 400.13 and ^13^C 100.6 MHz; CD_3_OD included TMS (*v*/*v*, 0.05%) as reference standard. All chemical shifts (δ) are in ppm and coupling constants (J) are given in Hertz (Hz). Doublet (d), and doublet of doublet (dd). Heteronuclear (^1^H-^13^C) single quantum correlation (HSQC). Long-range (^1^H-^13^C) Heteronuclear multiple bond correlation (^L-R^J_H-C_ HMBC).

**Structural detail for 3- and 5-*O*-caffeoylquinic acids (3, 4)**


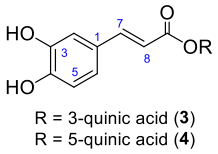


| **Experimental data in comparison with Ref.**^2^ | | | | |
| --- | --- | --- | --- | --- |
| **Metabolite** | **Position** | **δ_H_ (J in Hz)** | **δ_C_ (HSQC)** | (**^LR^J_H-C_**) **HMBC** |
| 3- and 5-*O*-caffeoylquinic acid (**3,4**) | 1 | - | 127.8 | - |
|  | 2 | 7.06 (d = 2.0) in **3** | 115.1 | 147.0; 149.5; 123.0 |
|  |  | 7.04 (d = 2.0) in **4** |  |  |
|  | 3 | - | 147.0 | - |
|  | 4 | - | 149.5 | - |
|  | 5 | 6.78 (d = 8.2) | 116.4 | 127.8; 147.0; 149.5; 123.0 |
|  | 6 | 6.96 (dd = 8.2; 2.0) | 123.0 | 115.1; 147.0; 149.5 |
|  | 7 | 7.59 (d = 15.9) in **3** | 146.8 | 115.1; 123.0; 169.0 |
|  |  | 7.55 (d = 15.9) in **4** |  |  |
|  | 8 | 6.30 (d = 15.9) in **3** | 115.1 | 127.8; 169.0 |
|  |  | 6.27 (d = 15.9) in **4** |  |  |
|  | 9 | - | 169.0 | - |

**Table S3.** ^1^H HR-MAS and 2D NMR-based structure confirmation of 3-*O*-caffeoylquinic acid and 5-*O*-caffeoylquinic acid (**3,4**) detected in the leaves of *Citronella gongonha* Mart. (Cardiopteridaceae). Experimental data: ^1^H 400.13 and ^13^C 100.6 MHz; CD_3_OD included TMS (*v*/*v*, 0.05%) as reference standard. All chemical shifts (δ) are in ppm and coupling constants (J) are given in Hertz (Hz). Doublet (d), doublet of doublet (dd). Heteronuclear (^1^H-^13^C) single quantum correlation (HSQC). Long-range (^1^H-^13^C) Heteronuclear multiple bond correlation (^L-R^J_H-C_ HMBC).

**Structural detail for kingiside (5)**

| **Experimental data in comparison with Refs.**^3,4^ | | | | |
| --- | --- | --- | --- | --- |
| **Metabolite** | **Position** | **δ_H_ (J in Hz)** | **δ_C_ (HSQC)** | (**^LR^J_H-C_**) **HMBC** |
| Kingiside (**5**) | 1 | 5.65 (d = 6.1) | 94.4 | 28.0; 76.5; 100.0 |
|  | 3 | 7.52 (s) | 154.1 | 94.4; 111.6; 28.0; 168.1 |
|  | 4 | - | 111.6 | - |
|  | 5 | 3.22 (m) | 28.0 | 154.1; 111.6; 174.5; 76.5; 40.0 |
|  | 6a, b | 3.01 (dd = 17.1; 7.6) | 34.4 | 111.6; 28.0; 174.5; 40.0 |
|  |  | 2.61 (17.1; 6.0) |  |  |
|  | 7 | - | 174.5 | - |
|  | 8 | 4.76 (dd = 6.6; 4.0) | 76.5 | 19.0 |
|  | 9 | 2.42 (dddd = 13.6; 10.2; 6.1; 4.1) | 40.0 | 94.4; 111.6; 28.0; 174.5 |
|  | 10 | 1.52 (d = 6.8) | 19.0 | 174.5; 76.5; 40.0 |
|  | 11 | - | 168.1 | - |
|  | 12 | 3.71 (s) | 51.5 | 168.1 |
|  | 1′ | 4.87 (br, d) | 100.0 | 94.4; 77.8; 75.2 |

**Table S4.** ^1^H HR-MAS and 2D NMR-based structure confirmation of kingiside (**5**) detected in the leaves of *Citronella gongonha* Mart. (Cardiopteridaceae). Experimental data: ^1^H 400.13 and ^13^C 100.6 MHz; CD_3_OD included TMS (*v*/*v*, 0.05%) as reference standard. All chemical shifts (δ) are in ppm and coupling constants (J) are given in Hertz (Hz). Singlet (s), doublet (d), doublet of doublets (dd), doublet of doublet of doublets (ddd), multiplet (m). Heteronuclear (^1^H-^13^C) single quantum correlation (HSQC). Long-range (^1^H-^13^C) Heteronuclear multiple bond correlation (^L-R^J_H-C_ HMBC).

**Structural detail for 8-epi-kingisidic acid (6)**

| **Experimental data in comparison to Ref.**^4^ | | | | |
| --- | --- | --- | --- | --- |
| **Metabolite** | **Position** | **δ_H_ (J in Hz)** | **δ_C_ (HSQC)** | (**^LR^J_H-C_**) **HMBC** |
| 8-epi-kngisidic acid (**6**) | 1 | 5.49 (d = 7.7) | 96.1 | 98.1 |
|  | 3 | 7.51 (s) | 154.1 | 96.1; 111.6; 28.0; 168.2 |
|  | 4 | - | 111.6 | - |
|  | 5 | 3.11 (m) | 28.0 | 96.1; 111.6 |
|  | 6a, b | 3.01 (dd = 17.1; 7.6) | 34.4 | 111.6; 28.0; 174.5; 40.0; |
|  |  | 2.61 (dd = 17.1; 6.0) |  |  |
|  | 7 | - | 174.5 | - |
|  | 8 | 4.76 (dd = 6.6; 4.0) | 76.5 | 19.0 |
|  | 9 | 2.42 (dddd = 13.6; 10.2; 6.1; 4.1) | 40.0 | 174.5 |
|  | 10 | 1.52 (d = 6.8) | 19.0 | - |
|  | 11 | - | 168.2 | - |
|  | 1′ | 4.48 (d = 7.8) | 98.1 | - |

**Table S5.** ^1^H HR-MAS and 2D NMR-based structure confirmation of 8-epi-kingisidic acid (**6**) detected in the leaves of *Citronella gongonha* Mart. (Cardiopteridaceae). Experimental data: ^1^H 400.13 and ^13^C 100.6 MHz; CD_3_OD included TMS (*v*/*v*, 0.05%) as reference standard. All chemical shifts (δ) are in ppm and coupling constants (J) are given in Hertz (Hz). Singlet (s), doublet (d), doublet of doublet (dd), multiplet (m). Heteronuclear (^1^H-^13^C) single quantum correlation (HSQC). Long-range (^1^H-^13^C) Heteronuclear multiple bond correlation (^L-R^J_H-C_ HMBC).

**Structural detail for (7*α*)-7-*O*-methylmorroniside (7)**


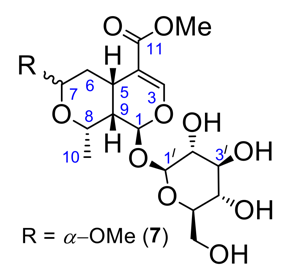


| **Experimental data in comparison with Ref.**^5^ | | | | |
| --- | --- | --- | --- | --- |
| **Metabolite** | **Position** | **δ_H_ (J in Hz)** | **δ_C_ (HSQC)** | (**^LR^J_H-C_**) **HMBC** |
| (7*α*)-7-*O*-methylmorroniside (**7**) | 1 | 5.83 (d = 9.3) | 95.5 | 74.0; 100.0 |
|  | 3 | 7.48 (br, s) | 153.5 | 111.6; 31.6; 168.2 |
|  | 4 | - | 111.6 | - |
|  | 5 | 2.80 (m) | 31.6 | - |
|  | 6a, b | 1.18 (dt = 13.0; 10.0) | 37.4 | - |
|  |  | 2.07 (m) |  |  |
|  | 7 | - | - | - |
|  | 7 – OCH_3_ | 3.50 (s) | - | - |
|  | 8 | 3.91 (m) | 74.0 | - |
|  | 9 | 1.80 (m) | 40.0 | - |
|  | 10 | 1.39 (d = 6.9) | 19.7 | 74.0; 40.0 |
|  | 11 | - | 168.2 | - |
|  | 11 – OCH_3_ | 3.69 (s) | 51.6 | - |
|  | 1′ | 4.87 (m) | 100.0 | - |

**Table S6.** ^1^H HR-MAS and 2D NMR-based structure confirmation of (7*α*)-7-*O*-methylmorroniside (**7**) detected in the leaves of *Citronella gongonha* Mart. (Cardiopteridaceae). Experimental data: ^1^H 400.13 and ^13^C 100.6 MHz; CD_3_OD included TMS (*v*/*v*, 0.05%) as reference standard. All chemical shifts (δ) are in ppm and coupling constants (J) are given in Hertz (Hz). Singlet (s), doublet (d), multiplet (m). Heteronuclear (^1^H-^13^C) single quantum correlation (HSQC). Long-range (^1^H-^13^C) Heteronuclear multiple bond correlation (^L-R^J_H-C_ HMBC).

**Structural detail for (7*β*)-7-*O*-methylmorroniside (8)**


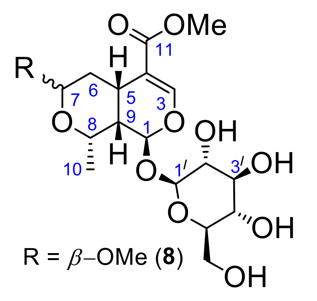


| **Experimental data in comparison with Ref.**^5^ | | | | |
| --- | --- | --- | --- | --- |
| **Metabolite** | **Position** | **δ_H_ (J in Hz)** | **δ_C_ (HSQC)** | (**^LR^J_H-C_**) **HMBC** |
| (7*β*)-7-*O*-methylmorroniside (**8**) | 1 | 5.87 (d = 9.2) | 95.5 | 99.9 |
|  | 3 | 7.51 (s) | 153.7 | 95.5; 111.6; 28.0; 168.1 |
|  | 4 | - | 111.6 | - |
|  | 5 | 3.11 (m) | 28.0 | 111.6; 99.9 |
|  | 6a, b | 1.52 (d = 6.8) | 34.3 | 40.3 |
|  |  | 1.92 (m) |  |  |
|  | 7 | 4.76 (m) | 99.9 | - |
|  | 7 – OCH_3_ | 3.37 (s) | 51.5 | - |
|  | 8 | 4.55 (m) | 65.6 | 99.9 |
|  | 9 | 1.82 (m) | 40.3 | - |
|  | 10 | 1.35 (d = 6.9) | 22.2 | - |
|  | 11 | - | 168.1 | - |
|  | 11 – OCH_3_ | 3.70 (s) | 51.6 | 168.1 |
|  | 1′ | 4.65 (d = 7.8) | 99.9 | - |

**Table S7.** ^1^H HR-MAS and 2D NMR-based structure confirmation of (7*β*)-7-*O*-methylmorroniside (**8**) detected in the leaves of *Citronella gongonha* Mart. (Cardiopteridaceae). Experimental data: ^1^H 400.13 and ^13^C 100.6 MHz; CD_3_OD included TMS (*v*/*v*, 0.05%) as reference standard. All chemical shifts (δ) are in ppm and coupling constants (J) are given in Hertz (Hz). Singlet (s), doublet (d), doublet of doublets (dd), multiplet (m). Heteronuclear (^1^H-^13^C) single quantum correlation (HSQC). Long-range (^1^H-^13^C) Heteronuclear multiple bond correlation (^L-R^J_H-C_ HMBC).

**Structural detail for alpigenoside (9)**

| **Experimental data in comparison with Ref.**^5^ | | | | |
| --- | --- | --- | --- | --- |
| **Metabolite** | **Position** | **δ_H_ (J in Hz)** | **δ_C_ (HSQC)** | (**^LR^J_H-C_**) **HMBC** |
| Alpigenoside (**9**) | 1 | 5.71 (d = 8.5) | 97.6 | 100.0 |
|  | 3 | 7.48 (br, s) | 153.4 | 97.6; 111.6; 31.6; 168.8 |
|  | 4 | - | 111.6 | - |
|  | 5 | 3.23 (m) | 31.6 | 175.0 |
|  | 6a, b | 2.42 (dddd = 13.6; 10.2; 6.1; 4.1) | 37.8 | 111.6; 31.6; 175.0; 46.0 |
|  |  | 2.81 (m) |  |  |
|  | 7 | - | 175.0 | - |
|  | 7 – OCH_3_ | 3.67 (s) | 51.5 | - |
|  | 8 | 4.04 (m) | 68.0 | - |
|  | 9 | 1.90 (m) | 46.0 | 97.6; 68.0; 22.2 |
|  | 10 | 1.35 (d = 6.9) | 22.2 | 68.0; 46.0 |
|  | 11 | - | 168.8 | - |
|  | 11 – OCH_3_ | 3.69 (s) | 51.5 | 168.8 |
|  | 1′ | 4.87 (m) | 100.0 | - |

**Table S8.** ^1^H HR-MAS and 2D NMR-based structure confirmation of alpigenoside (**9**) investigated in the leaves of *Citronella gongonha* Mart. (Cardiopteridaceae). Experimental data: ^1^H 400.13 and ^13^C 100.6 MHz; CD_3_OD included TMS (*v*/*v*, 0.05%) as reference standard. All chemical shifts (δ) are in ppm and coupling constants (J) are given in Hertz (Hz). Singlet (s), doublet (d), multiplet (m). Heteronuclear (^1^H-^13^C) single quantum correlation (HSQC). Long-range (^1^H-^13^C) Heteronuclear multiple bond correlation (^L-R^J_H-C_ HMBC).

**References**

1. Ali, S., Rech, K. S., Badshah, G., Soares, F. L. F. & Barison, A. ^1^H HR-MAS NMR-Based Metabolomic Fingerprinting to Distinguish Morphological Similarities and Metabolic Profiles of *Maytenus ilicifolia*, a Brazilian Medicinal Plant. *J Nat Prod* **84**, 1707–1714 (2021).

2. Ali, S. *et al.* High-Resolution Magic Angle Spinning (HR-MAS) NMR-Based Fingerprints Determination in the Medicinal Plant *Berberis laurina*. *Molecules* **25**, 3647 (2020).

3. Bailleul, F., Leveau, A. M. & Durand, M. Nouvel Iridoide des Fruits de *Lonicera alpigena*. *J Nat Prod* **44**, 573–575 (1981).

4. Ma, W.-G., Fuzzati, N., Wolfender, J.-L., Hostettmann, K. & Yang, C.-R. Rhodenthoside A, a New Type of Acylated Secoiridoid Glycoside from *Gentiana rhodentha*. *Helv Chim Acta* **77**, 1660–1671 (1994).

5. Hu, J.-F. *et al.* Secoiridoid Glycosides from the Pitcher Plant *Sarracenia alata*. *Helv Chim Acta* **92**, 273–280 (2009).
